# Standardized aqueous extract of *Abutilon theophrasti* Medic. ameliorates oxidative stress and inflammatory responses against hydrochloric acid/ethanol-induced gastric ulcer in rats

**DOI:** 10.3389/fphar.2025.1599810

**Published:** 2025-06-18

**Authors:** Hui Guo, Zihan Xu, Lili Zhu, Meng Zhu, Weijin Zhang, Man Gong, Mengyun Liu, Mengmeng Wang, Erping Xu, Liping Dai

**Affiliations:** ^1^ Henan University of Chinese Medicine (HUCM), Collaborative Innovation Center of Research and Development on the Whole Industry Chain of Yu-Yao, Henan Province, Zhengzhou, Henan, China; ^2^ Schoolof Pharmacy, HUCM, Zhengzhou, Henan, China

**Keywords:** *Abutilon theophrasti* Medic., gastric ulcer, bioinformatics, RAW264.7 cells, ROS/Akt/NF-κB

## Abstract

**Background:**

The herb *Abutilon theophrasti* Medic. (AT, *Qingma* in Chinese), a widely distributed medicinal plant in various regions worldwide, is commonly used in China for treating inflammatory diseases of the gastrointestinal (GI) tract, such as dysentery. However, the pharmacological basis of this herb for treating peptic ulcer, also an inflammatory condition in the GI tract, remains insufficiently understood.

**Purpose:**

The aim of this study is to investigate the ameliorating effects of a standardized aqueous extract of AT (ATAE) on experimental gastric ulcer (GU) in rats and explore whether the human GU-associated molecules/signaling pathways obtained using bioinformatics have a role to play in the drug’s efficacy for molecular mechanism elucidation.

**Methods:**

Ultra-performance liquid chromatography (UPLC) and UPLC coupled to tandem mass spectrometry (UPLC-MS) were used for standardization of ATAE. GSE233973 and GSE264263 datasets with *Helicobacter pylori* (HP)*-*infected and normal human biospecimens from the Gene Expression Omnibus (GEO) database were normalized and subjected to identification of differentially expressed genes (DEGs). A protein–protein–substance network construction and Gene Ontology (GO) and Kyoto Encyclopedia of Genes and Genomes (KEGG) pathway enrichment analyses were performed to explore and visualize the biological targets, effective substances, and signaling pathways involved in the anti-GU effects of ATAE. Hydrochloric acid/ethanol (HCl/EtOH)-induced GU rats and lipopolysaccharide (LPS)-stimulated RAW264.7 cells were used as models to investigate the GU-ameliorating effects and underlying mechanism of ATAE. Multiple bioassays/kits were employed to observe histopathological changes and expression/production levels of cytokines/molecules.

**Results:**

A total of 26 compounds were identified in ATAE, comprising 10 flavonoids, 7 organic acids, 5 amines, and 4 other compounds. The content of rutin in ATAE was 0.38%. *In vivo*, ATAE markedly attenuated the HCl-/EtOH-induced GU and mucosal injuries; decreased the levels of pro-inflammatory/oxidant mediators, including TNF-α, IL-1β, IL-6, and malondialdehyde (MDA); increased the anti-oxidant activity of mediators superoxide dismutase (SOD) and glutathione peroxidase (GSH-Px); and inhibited the phosphorylation/activation of Akt, IκBα, and NF-κB p65 in gastric tissues. *In vitro,* ATAE treatment significantly lowered nitric oxide (NO) and reactive oxygen species (ROS) production, attenuated nuclear translocation of NF-κB p65, and modulated mRNA expression levels of NF-κB-regulated mediators, including TNF-α, IL-1β, IL-6, MnSOD, GSH-Px, CAT, VCAM-1, and MMP-9. For the bioinformatics study, 24 hub genes were screened and found to be predominantly enriched in oxidative stress- and inflammatory response-associated pathways; quercetin and caffeic acid were identified as the most effective substances responsible for ATAE’s anti-GU effects. Overall, the presented results greatly supported and validated the essential inflammation and oxidation events implicated in the mechanistic investigation using bioinformatics.

**Conclusion:**

ATAE ameliorates oxidative stress and inflammatory responses against HCl/EtOH-induced GU in rats, which are probably associated with inhibiting the ROS/Akt/NF-κB signaling pathway. The novel findings of this study, for the first time, provide scientific justifications for the use of AT in treating peptic ulcer. Future studies are warranted to elucidate the clinical potential and a more comprehensive understanding of the underlying mechanisms of AT in treating GU.

## 1 Introduction

Gastric ulcer (GU) is one of the most common digestive disorders with a high lifetime prevalence of approximately 5%–10% in humans and is known to degrade the quality of life (Gong et al., 2022; Li et al., 2023). Current therapies for GU include antacids, H2 receptor blockers (e.g.*,* ranitidine and famotidine), and proton pump inhibitors (e.g., omeprazole and lansoprazole). Long-term use of these treatments is always accompanied by adverse effects, including drug interactions, increased risk of gastrointestinal infections, and potential kidney impairment (Gong et al., 2024; Gupta et al., 2023; Maarouf and Jones, 2020). Therefore, safe and effective therapies are urgently needed to be implemented.

The herb *Abutilon theophrasti* Medic. (AT, *Qingma* in Chinese) is an annual herbaceous plant and widely distributed in various regions worldwide. Traditionally, AT has been employed medicinally in China and was initially recorded in *Newly Revised Materia Medica*, a formal pharmacopoeia published during the Tang Dynasty (A.D. 618–907) (Jia et al., 2024). Generally, the whole herb or leaves are primarily harvested and used as AT/*Qingma* and distinct from another well-known medicine *Qingmazi* (i.e., AT’s seeds) harvested at different times (Tian et al., 2018). According to traditional Chinese medicine (TCM) theory, AT is bitter in flavor and neutral in property, with functions of detoxifying and dispelling wind cold, and is particularly associated with the spleen and stomach meridians, indicating its potential for treating/benefiting digestive disorders (NJUCM, 2014; Tian et al., 2012). In clinics, AT is widely used for treating inflammatory disorders of the gastrointestinal tract (e.g., dysentery). Despite different causes and symptoms, dysentery and GU are both inflammatory conditions of the gastrointestinal tract. However, whether AT could be used as an anti-GU agent remains to be addressed. As phytochemical studies reported, the ingredients in AT are mainly flavonoids (e.g., rutin and quercetin) and organic acids (e.g., caffeic acid) (Tian et al., 2014; Tian et al., 2019), with antioxidant, antibacterial, and anti-inflammatory activities, and have been reported to exert therapeutic effects in treating GU (Akash et al., 2024; Salem et al., 2024), suggesting the potentials of compounds/herb for use as agents for GU treatment.

In bioinformatics research, the Gene Expression Omnibus (GEO) database hosts a vast collection of gene expression data from diverse biological samples, including those from patients with specific diseases or human cells with specific treatments, which facilitates comprehensive analysis and the identification of differentially expressed genes (DEGs) (Alameer and Chicco, 2022; Karri et al., 2024). Then, by combining GEO data with routine network pharmacology, researchers can construct protein–protein interaction (PPI) networks and perform functional enrichment analyses. Accordingly, a bioinformatics study using GU-associated GEO datasets integrated with network pharmacology aims to provide a comprehensive mechanistic understanding of AT in managing GU.

In this study, we investigated whether a standardized aqueous extract of AT (ATAE) ameliorates hydrochloric acid/ethanol (HCl/EtOH)-induced experimental GU in rats and explored the involvement of human GU-associated molecules/signaling pathways in the drug’s efficacy. The findings of this work could provide scientific justifications for the use of AT in treating peptic ulcer.

## 2 Materials and methods

### 2.1 Chemicals and reagents

Rutin (purity ≥98%) was purchased from Zhibiao Huachun Biotechnology Co., Ltd (Chengdu, China). Lipopolysaccharide (LPS) from *Escherichia coli* O55:B5, dimethyl sulfoxide (DMSO), and 3-(4,5-dimethylthiazolyl-2)-2,5-diphenyltetrazolium bromide (MTT) were obtained from Sigma Chemicals Ltd (St. Louis, United States). Penicillin, streptomycin, Dulbecco’s modified Eagle medium (DMEM), bovine serum albumin (BSA), and fetal bovine serum (FBS) were purchased from Hyclone (Logan, United States). Superoxide dismutase (SOD), malondialdehyde (MDA), and glutathione peroxidase (GSH-Px) detection test kits were purchased from Nanjing Jiancheng Bioengineering Institute (Nanjing, China). Griess regents and staining reagents, including periodic acid–Schiff (PAS) and hematoxylin and eosin (H&E), were purchased from Solarbio Science and Technology Co., Ltd. (Beijing, China). Enzyme-linked immunosorbent assay (ELISA) kits for determining levels of TNF-α, IL-6, and IL-1β in gastric tissues from rats were obtained from Darwin Biotechnology Co., Ltd. (Guangzhou, China). HPLC-grade acetonitrile (ACN) was obtained from RCI Labscan Limited (RCI, Thailand). Purified water for HPLC analysis and sample preparation was prepared using a Milli-Q system (Millipore, United States). Other materials used in the bioassay were purchased from Life Technologies Inc (GIBICO, United States). Monoclonal antibodies against Akt, phospho-Akt (Ser473), IκBα, phospho-IκBα (Ser32), NF-κB p65, phospho-NF-κB p65(Ser536), β-actin, and HRP-linked secondary antibodies anti-rabbit IgG were provided by Cell Signaling Technology (Boston, United States).

### 2.2 Preparation and standardization of ATAE

The whole raw herb of *A. theophrasti* was collected in Xinzheng, Henan Province, China, with geographical coordinates of 113°E longitude and 34°N latitude. Professor Dai Liping from Henan University of Chinese Medicine (HUCM) confirmed its authenticity. A voucher specimen, designated as No.20200624, has been archived at the R&D Center for Classic TCM Formulas and Wellness Products, HUCM.

AT is traditionally used in decoction (extracted using water). To be consistent with traditional usage, an ATAE was prepared for use in this study. AT was ground and macerated for 1 h with purified water (1:10, w/v) at room temperature and then refluxed twice (1 h each time). After cooling, the extracts were filtered and combined. The filtrate was then concentrated under reduced pressure, followed by freeze-drying to obtain ATAE with a calculated yield of 15.19% (w/w).

### 2.3 Ultra-performance liquid chromatography (UPLC) analyses of ATAE

For the quality control of ATAE, an ultra-performance liquid chromatography (UPLC) analysis was performed on a Vanquish UHPLC system (Thermo Fisher SCIENTIFIC, United States) using a previously reported method with minor modifications (Tian et al., 2014). In details, the separations were performed using a Thermo Scientific C_18_ analytical column (150 mm × 2 mm I.D., 2.5 µm). A linear gradient system consisted of A (0.1% phosphoric acid in purified water) and B (ACN). The gradient elution profile was as follows: 0–10 min, 5%–15% B; 10–20 min, 15% B. The flow rate was maintained at 0.3 mL/min, and the column temperature was set at 30°C. The chromatograms were monitored with the DAD detector at a wavelength of 328 nm. ATAE or chemical standard of rutin was dissolved in 80% methanol and then filtered through a 0.45-µm PVDF filter membrane. Ten microliters of each filtrate was injected into the UPLC system for analysis.

### 2.4 UPLC coupled with tandem mass spectrometry (UPLC-MS) analyses

The UPLC-Oribtrap-Exploris-120-MS liquid chromatography–mass spectrometry system (Thermo Scientific, Waltham, MA, United States) was used to obtain the chemical profiles of ATAE. The separation was achieved using A HYPERSILGOLD Vanquish C_18_ (2.1 mm × 100 mm, 1.9 μm) column. The mobile phase consisted of ACN (A) and 0.1% aqueous formic acid (B), with a flow rate of 0.3 mL/min. The gradient elution condition was set as follows: 0–5 min, 5%–10% A; 5–10 min, 10%–14% A; 10–13 min, 14%–20% A; 13–18 min, 20%–45% A; 18–25 min, 45% A; 25–30 min, 45%–70% A; 30–35 min, 70% A. The injection volume of the aforementioned test sample for ATAE in Section 2.3 was 1 µL, and the column temperature was maintained at 30°C.

MS data were acquired in fast chromatography MS^2^ mode with the instrument working in both negative ion (ESI^−^) and positive ion (ESI^+^) modes. The experimental parameters were configured as follows: the spray voltage was 2.5 kV for the negative ion mode and 3.5 kV for the positive ion mode; the isolation window was set at m/z 2; the RF Lens% was 70; the sheath gas pressure was maintained at 45 Arb; the auxiliary gas pressure was kept at 15 Arb; the sweep gas pressure was 0 Arb; the capillary and vaporizer temperatures were set to 320°C and 350°C, respectively; the scanning range was m/z 100–1000; the stepped normalized collision energies (NCEs) were applied at 15, 30, and 45 eV.

### 2.5 Bioinformatics analyses

#### 2.5.1 GU-associated gene collection

GSE233973 and GSE264263 datasets were downloaded from the GEO database. All samples in these datasets were derived from human tissues/cells. The GSE233973 involved 13 samples of gastric mucosa with *Helicobacter pylori* (HP)-infected gastritis and 9 samples of normal gastric mucosa. The GSE264263 included three samples of HP-infected AGS cells (a gastric carcinoma cell line) and three samples of uninfected AGS cells. Multi-chip joint analysis and batch normalization were performed using an online tool NetworkAnalyst (https://www.networkanalyst.ca). For the detection of DEGs, the thresholds were set with |logFC| > 1 and *p* < 0.05. The volcano plots from Sangerbox 3.0 (http://sangerbox.com/) were used for data analysis and visualization. Additional GU-associated genes with duplicates removed were compiled from well-known databases including GeneCards, OMIM, and TTD.

#### 2.5.2 Substance–gene intersection analyses

Following chemical profile analyses of ATAE in the above section, the targeting genes for each identified analyst were collected using online databases including TCMSP, PharmMapper, and SwissTargetPrediction. Subsequently, the genes obtained were visualized in a Venn diagram at Bioinformatics (https://www.bioinformatics.com.cn).

#### 2.5.3 Protein–protein–substance interaction network construction

Genes at the intersection of the aforementioned datasets were imported into the STRING database (http://string-db.org), with the species filter set to “*Homo sapiens*” on the interface. For network construction and systematic analysis to identify key genes, Cytoscape 3.10.1 was used. Key genes were determined based on degree values, with gene importance visually represented through icon size and color variations. Afterward, the interactions of the substances with the hub genes (arranged in the two inner layers) were merged into the PPI network and shown in the outer layer, and icon for substance in size was displayed based on its degree value. Additionally, a heatmap plot with clustering analyses was carried out for visualizing complex gene expression data and validating top genes using Bioinformatics tools.

#### 2.5.4 GO and KEGG enrichment analyses

Enrichment analyses for Gene Ontology (GO) and Kyoto Encyclopedia of Genes and Genomes (KEGG) were performed using the DAVID online tool (https://david.ncifcrf.gov/). GO chord plot at SangerBox and Sankey and dot plot at Bioinformatics were used for visualization of GO and KEGG analyses, respectively.

### 2.6 Animal experiments

Forty male Wistar rats, aged between 3 and 8 weeks, with an average body weight of 180 ± 20 g, were procured from Jinan Pengyue Experimental Animal Breeding Co., Ltd., with the license number SYXK (Lu) 20190003. The protocols for animal care and manipulation were sanctioned by the Animal Ethics Committee of HUCM, with the approval number DWLL202009432.

#### 2.6.1 Experiment grouping

After 1 week of habituation, rats were randomly divided into five groups (eight rats/group): normal control (CON) group, model (MOD) group, pretreatment dose groups of ATAE (50 and 100 mg/kg for ATAE-L and ATAE-H, respectively), and positive drug group treated with ranitidine (35 mg/kg; RAN). The sample size for each experimental group was calculated based on the results of a pilot study conducted using G*Power software (version 3.1.9.2 from Kiel University), as previously detailed (Chen et al., 2023). The study adopted a significance level of 5% (α = 0.05) for a two-tailed test and a power level of 80% (1 − β = 0.8). In the pilot study, which involved two groups with n = 3 (illustrated in Supplementary Figure S1), the *Ulcer index*, an index for measuring GU injury (explained in a subsequent section), was 16.21% for the model group and 9.64% for the ATAE-H (100 mg/kg) group. The corresponding standard deviations (SDs) were 4.86% and 3.72%, respectively. The calculated minimum sample size for each group was 8. Accordingly, we set the sample size to 8. The dose of ATAE-H (100 mg/kg) for rats is equivalent to an adult consuming approximately 15 g/day, which aligns with the clinical recommendation of a daily dose of AT ranging from 10 to 30 g for humans (NJUCM, 2014).

#### 2.6.2 Drug treatment

For each group, rats were administered intragastrically with a uniform volume of 1% CMC-Na solution, acting as the vehicle. To obtain the CMC-Na solution, 1% (w/v) CMC-Na was dissolved in hot water (60°C–70°C) under stirring and then cooled to room temperature for complete hydration. The RAN and ATAE groups received the solution with the drug, whereas the NOR and MOD groups were given the solution without the drug. This procedure was conducted twice daily at 8 a.m. and 8 p.m. for 3 consecutive days. After the final administration of the drugs, the rats were subjected to an overnight fast while still having unrestricted access to water.

#### 2.6.3 GU modeling

On day 4, 30 min after the final intragastric administration of drugs, rats except for those in the CON group were administrated with 150 mM HCl/EtOH solution (10 mL/kg). As reported, the HCl/EtOH solution was prepared as follows: 150 mmol HCl was taken and dissolved in 1 L of 60% (v/v) EtOH-H_2_O. Then, HCl/EtOH-induced GU modeling was established by fasting for 1 h (Gong et al., 2022; Hossen et al., 2019; Li et al., 2023). Following euthanasia *via* carbon dioxide inhalation, gastric tissues were harvested, washed, and then either immersed in 10% neutral-buffered formalin for fixation or flash-frozen in liquid nitrogen for subsequent analyses.

### 2.7 Ulcer index analysis

After being rinsed with cooled saline, the stomach tissue was carefully unfurled and photographed using a digital camera. The area (mm^2^) of each mucosal erosive lesion was then quantified using ImageJ software, and then, the ulcer index (%) was calculated using the following formula (Feng et al., 2023).

$$\text{Ulcer index }\left(\%\right)=\frac{\text{gastric}\;\text{mucosal}\;\text{erosive}\;\text{lesion}\;\text{area}}{\text{corresponding}\;\text{to}\;\text{the}\;\text{total}\;\text{area}\;\text{of}\;\text{stomach}\;\text{tissue}}\times 100\%.$$

### 2.8 Histological analysis

Sections of each fixed gastric tissue sample, 5 μm in thickness, were prepared and subjected to H&E and PAS staining to observe histopathological and glycoprotein alterations in the gastric mucosa, respectively. Additionally, the gastric histopathological score and the mucus area (i.e., PAS-positive area) ratio were determined using the methods previously described (Li et al., 2023).

### 2.9 Biochemical analysis

Biochemical assays were conducted to measure the concentrations of IL-1β, IL-6, TNF-α, SOD, MDA, and GSH-Px in the gastric tissues, following the protocols provided by the respective kit manufacturers.

### 2.10 Cell culture

RAW264.7 cells were obtained from the American Type Culture Collection (Manassas, VA, United States) and cultured in DMEM containing 5% heat-inactivated FBS and 1% antibiotics of penicillin/streptomycin at 37°C under humidified 5% CO_2_ in air.

### 2.11 Cell viability assay

Cells were plated in 96-well plates at a density of 5 × 10^3^ cells/well and left to adhere overnight. Following this, the cells were exposed to various concentrations of ATAE for 1 h, after which they were cultured with or without LPS (2.0 μg/mL) for an additional 24 h. Cell viability was then evaluated using the MTT assay as described (Gerlier and Thomasset, 1986).

### 2.12 Nitric oxide (NO) production

Cells at a density of 2 × 10^5^ cells/mL were placed in 24-well plates and left to adhere overnight. Afterward, these cells were pretreated with indicated concentrations of ATAE for 1 h and then further incubated with 2 μg/mL of LPS for another 24 h. The supernatant from each well was harvested for the assessment of nitric oxide (NO) levels using the Griess regents (Zhang et al., 2024).

### 2.13 Reactive oxygen species (ROS) level

Reactive oxygen species (ROS) production was carried out using the fluorescent probe 2′,7′-dichlorofluorescein diacetate (DCFH-DA). Olympus IX73 inverted fluorescence microscope and ImageJ software were used to measure and analyze ROS fluorescence, respectively, as previously described (Zhang et al., 2024).

### 2.14 RNA extraction and quantitative real-time PCR (RT-qPCR)

Adhered cells (2 × 10^5^ cells/well) in 6-well plates were pretreated with ATAE (100 μg/mL) for 1 h and then cultured with 2 μg/mL LPS for another 6 h. Nucleic acid was purified from the gathered cellular material using TRIzol reagent, which was then converted into cDNA using the ScriptTM RT reagent Kit, procured from ServiceBio, China. Thereafter, the cDNA was analyzed through RT-qPCR with SYBR GREEN, supplied by Monad, CHINA (Wang C. et al., 2024). Data normalization was performed relative to GAPDH levels, and the analysis was conducted using the 2^−ΔΔCT^ method. The sequences of the primers are detailed in Table 1.

**TABLE 1:** Primer sequences.

Gene	Forward primer sequence (5′–3′)	Reverse primer sequence (5′–3′)
IL-1β	CAC​TAC​AGG​CTC​CGA​GAT​GAA​CAA​C	TGT​CGT​TGC​TTG​GTT​CTC​CTT​GTA​C
IL-6	CTC​CCA​ACA​GAC​CTG​TCT​ATA​C	CCA​TTG​CAC​AAC​TCT​TTT​CTC​A
TNF-α	ATG​TCT​CAG​CCT​CTT​CTC​ATT​C	GCT​TGT​CAC​TCG​AAT​TTT​GAG​A
MnSOD	CTG​TGG​GAG​TCC​AAG​GTT​CA	GAG​CAG​GCA​GCA​ATC​TGT​AAG
GSH-Px	ACA​GTC​CAC​CGT​GTA​TGC​CTT​C	CTC​TTC​ATT​CTT​GCC​ATT​CTC​CTG
CAT	TGT​GAA​CTG​TCC​CTT​CCG​TG	CGT​CTG​TTC​GGG​AGC​ACT​AA
VCAM-1	GCC​ACC​CTC​ACC​TTA​ATT​GCT​ATG	TGT​GCA​GCC​ACC​TGA​GAT​CC
MMP-9	GCC​CTG​GAA​CTC​ACA​CGA​CA	TTG​GAA​ACT​CAC​ACG​CCA​GAA​G
Nrf2	TCA​GCG​ACA​GAA​GGA​CTA​AG	AGG​CAT​CTT​GTT​TGG​GAA​TG
Keap1	AGT​GGC​ATC​CTG​CGT​TTC​T	CAA​CAC​CAC​ACC​AAC​ATT​A
β-actin	TGC​AGT​TGC​TTT​ATT​CTC​CCA​T	TGT​TGC​CAC​GCC​TTC​ATT​C

### 2.15 NF-κB p65 immunofluorescence staining

Cells (1 × 10^4^ cells/mL) were seeded and allowed to adhere overnight, then treated with ATAE for 1 h, followed by stimulation with LPS (2 μg/mL) for an additional 1 h. Then, the cells on the chamber slides were washed, fixed, permeabilized, and incubated with the NF-κB p65 primary antibody and then with the second antibody. Finally, the slides were rinsed and subjected to staining with 4′, 6′-diamidino-2-phenylindole (DAPI) to visualize the cell nuclei (Qiu et al., 2024).

### 2.16 Western blot analysis

Tissues from the stomach were homogenized in RIPA buffer, and protein levels were determined using a BCA protein assay kit. Western blotting was performed based on prior methods. The primary antibodies for the phosphorylated forms of Akt, IκBα, and NF-κB p65 were diluted at ratios of 1:2,000, 1:1,000, and 1:500, respectively. In contrast, the primary antibodies for their non-phosphorylated forms and β-actin were all diluted at a ratio of 1:1,000. Band intensities were analyzed using ImageJ 1.54 g software, with protein expression normalized against the housekeeping protein β-actin for each experiment (Guo et al., 2024).

### 2.17 Statistical analysis

Data analysis was conducted using GraphPad Prism 8.3 software (CA, United States). Results are presented as mean ± SEM (standard error of mean) of at least three determinations in triplicate. Statistical significance was ascertained using one-way ANOVA, supplemented by Tukey’s multiple comparisons test. *p < 0.05 was set as the threshold for statistical significance.

## 3 Results

### 3.1 Chemical analyses of ATAE

**FIGURE 1:**
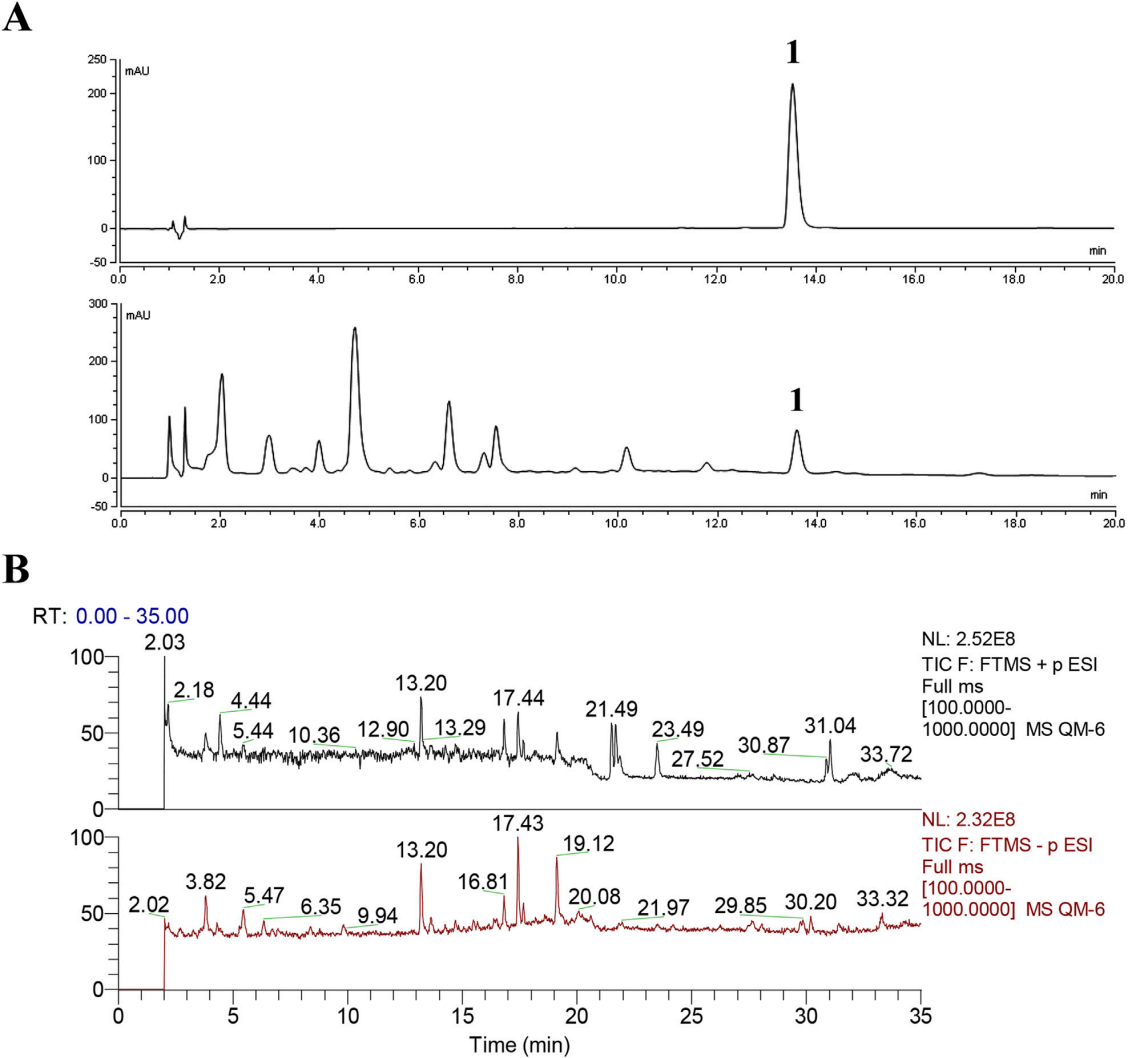
Chemical analyses of ATAE. **(A)** UPLC chromatograms of chemical standard rutin (Peak 1, upper panel) and ATAE (lower panel), and the content of rutin in ATAE is 0.38%. **(B)** Representative total ion chromatograms of ATAE in the positive (upper panel) and negative (lower panel) ion modes by using UPLC-MS^2^. Details on identified analytes are shown in Table 2.

Chemical profiles/standardization of the extract are indispensable for the bioinformatic and pharmacological study. For the standardization of ATAE, the content of rutin was determined using UPLC. Additionally, the chemical composition of ATAE was elucidated using UPLC-MS. As indicated in ATAE, the content of rutin (the single marker widely used for quality control of AT) was 0.38%, and the representative UPLC chromatogram is shown in Figure 1A; 26 analysts were tentatively identified, comprising 10 flavonoids, 7 organic acids, 5 amines, and 4 others; representative total ion chromatograms and analyte details are shown in Figure 1B and Table 2, respectively.

**TABLE 2:** Analytes identified in ATAE using UPLC-MS^2^ **.**

No.	TR (min)	Analyte	Formula	Adduct	Measured mass (m/z)	Theoretical mass (m/z)	Error (ppm)	MS^2^
1	3.35	Protocatechuic acid	C_7_H_6_O_4_	[M-H]^-^	153.0190	153.0193	−1.96	153.0190, 123.9022, 109.0296, 103.7952, 70.0907, and 46.6035
2	4.11	3-Indoleacrylic acid	C_11_H_9_NO_2_	[M + H]^+^	188.0700	188.0706	−3.46	170.0595, 146.0595, 144.0803, and 118.0647
3	5.38	2-(D-glucosyloxy)benzoic acid	C_13_H_16_O_8_	[M-H]^-^	299.0769	299.0772	−0.90	299.0769, 279.0676, 252.8285, 231.9310, 137.0244, 133.9888, and 79.5656
4	6.28	Fructose carboxylic acid	C_7_H_12_O_8_	[M-H]^-^	223.0454	223.0459	−2.20	205.0354, 176.8369, 150.2097, 85.0293, and 55.9875
5	6.70	Caffeic acid	C_9_H_8_O_4_	[M-H]^-^	179.0348	179.0350	−1.28	179.0348, 150.9542, 135.0452, 82.592, 77.2116, 65.047, and 53.6142
6	13.18	Myricitrine	C_21_H_20_O_12_	[M + H]^+^	465.1011	465.1028	−3.66	465.1011, 387.4034, 303.049, 256.3948, and 109.9037
7	13.24	Quercetin	C_15_H_10_O_7_	[M + H]^+^	303.0490	303.0499	−3.13	303.0490, 280.8672, 182.6891, 167.4775, 117.0427, and 65.8934
8	13.49	Rutin	C_27_H_30_O_16_	[M + H]^+^	611.1591	611.1607	−2.59	303.0490, 304.0523, 129.0538, 85.0284, and 71.0490
9	13.57	Quercetin-7-O-β-glucoside	C_21_H_20_O_12_	[M-H]^-^	463.0877	463.0882	−0.99	463.0877, 336.0646, 170.7721, 152.0185, 104.4786, and 76.6867
10	14.75	Apigenin-7-glucoside	C_21_H_20_O_10_	[M-H]^-^	432.1043	432.1056	−2.94	432.1043 and 269.0460
11	15.04	Kaempferol-3-O-β-glucoside	C_21_H_20_O_11_	[M + H]^+^	449.1070	449.1078	−1.87	449.1070, 287.0542, 172.8431, 133.7953, and 79.7426
12	17.43	Tiliroside	C_30_H_26_O_13_	[M-H]^-^	593.1300	593.1301	−0.22	593.1293, 240.0611, 197.4149, 163.8717, 138.2498, and 105.3292
13	18.52	Kaempferol	C_15_H_10_O_6_	[M-H]^-^	285.0400	285.0405	−1.93	285.0400 and 114.9110
14	19.12	Chrysin-5-O-glucoside	C_23_H_24_O_12_	[M-H]^-^	491.1190	491.1195	−1.04	401.0871, 248.4818, 210.784, 119.6924, 94.9649, and 72.0552
15	20.14	Butyl paraben	C_11_H_14_O_3_	[M-H]^-^	193.0867	193.0870	−1.61	193.0867, 137.5848, 99.2307, and 81.6786
16	20.21	Luteolin	C_15_H_10_O_6_	[M + H]^+^	287.0552	287.0550	0.57	260.4561, 226.2152, 221.2249, 169.1219, and 60.0557
17	20.79	2-Amino-1,3,4-octadecanetriol	C_18_H_39_NO_3_	[M + H]^+^	318.2991	318.3003	−3.68	318.0881
18	21.97	Dihomo-γ-linolenic acid	C_20_H_34_O_2_	[M-H]^-^	305.2463	305.2486	−7.53	305.2463 and 261.2588
19	28.72	α-Pyrrolidinopropiophenone	C_13_H_17_NO	[M + H]^+^	204.1376	204.1383	−3.28	204.1376
20	29.01	3,5-Di-tert-butyl-4-hydroxybenzaldehyde	C_15_H_22_O_2_	[M + H]^+^	235.1685	235.1693	−3.32	235.1685, 219.1374, 179.1062, 123.0436, and 57.0697
21	29.86	Glycyrrhetinic acid	C_30_H_46_O_4_	[M + H]^+^	471.3456	471.3469	−2.85	471.3454, 425.3414, 317.2093, 263.1622, 235.1686, 217.1578, 189.1636, and 95.0848
22	30.01	Diisobutylphthalate	C_16_H_22_O_4_	[M + H]^+^	279.1581	279.1591	−3.55	167.0340, 149.0229, and 57.0699
23	30.02	Monobutyl phthalate	C_12_H_14_O_4_	[M + H-H_2_O]^+^	205.0852	205.0859	−3.46	149.0230 and 57.0698
24	32.04	Hexadecanamide	C_16_H_33_NO	[M + H]^+^	256.2628	256.2635	−2.77	256.2636 and 257.2667
25	32.24	Oleamide	C_18_H_35_NO	[M + H]^+^	282.1783	282.1780	1.03	282.1780, 265.2515, 247.2416, 177.1634, 149.1317, 111.1163, 97.1009, and 83.0853
26	33.17	Erucamide	C_22_H_43_NO	[M + H]^+^	338.3405	338.3417	−3.69	303.30463, 195.17434, 163.14755, 149.1321, 121.10118, 111.11655, 97.10087, 83.08531, and 69.0696

### 3.2 ATAE ameliorated HCl/EtOH-induced experimental GU in rats

**FIGURE 2:**
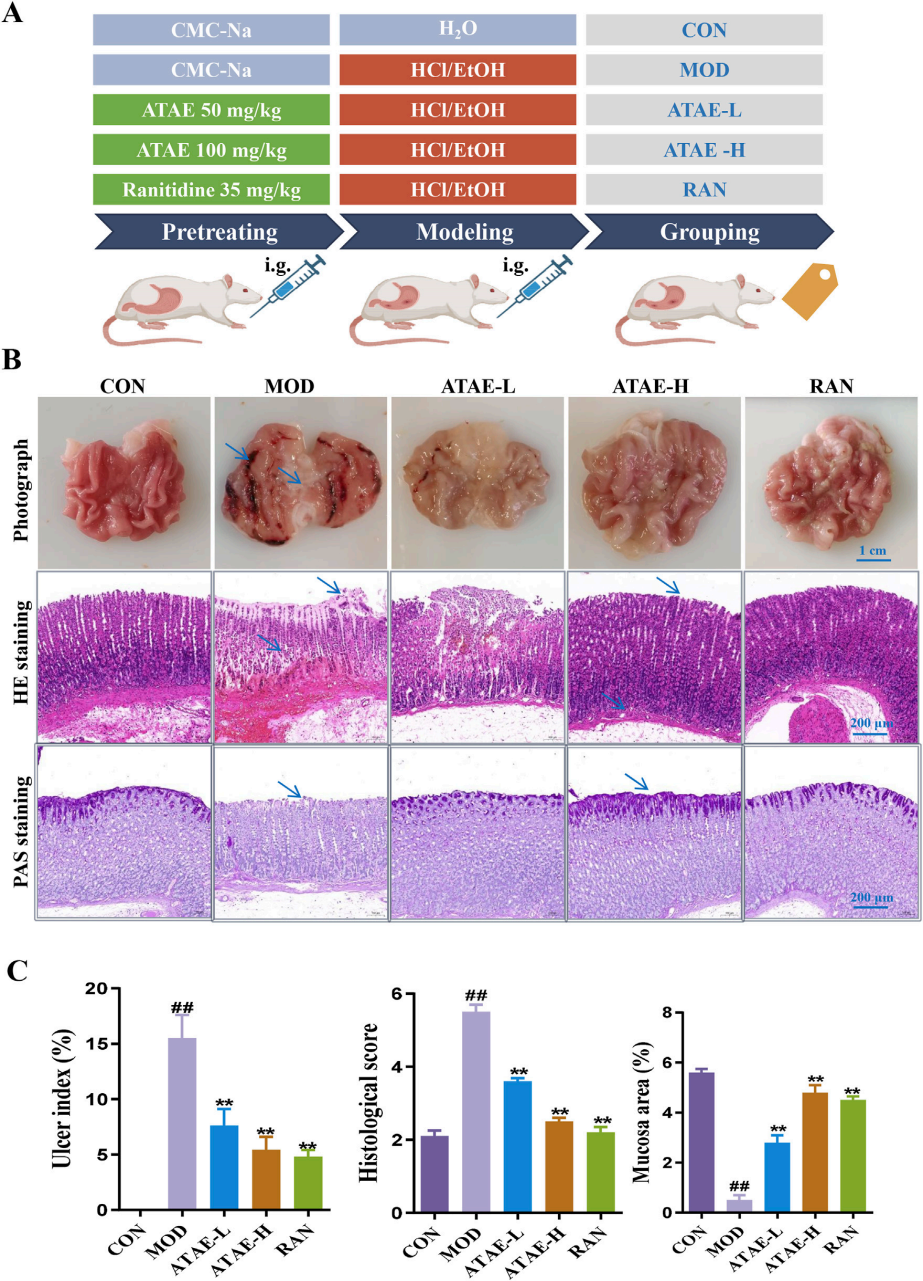
ATAE ameliorated HCl/EtOH-induced experimental GU in rats. **(A)** Experimental grouping. **(B)** Representative macroscopic images of the gastric tissue (upper panel, scale bar: 1 cm), hematoxylin and eosin (H&E) staining of gastric tissues (middle panel, scale bar: 200 μm), and periodic acid–Schiff (PAS) staining of gastric mucosa (lower panel, scale bar: 200 μm). **(C)** Statistical analyses on the ulcer index, histological score, and mucosa area (n = 8). Data are expressed as mean ± SEM. ##*p* < 0.01 vs CON group; *p < 0.05 and **p < 0.01 vs MOD group.

The HCl/EtOH-induced GU model exhibits characteristics akin to human ulcerative conditions, making it an ideal model for assessing the anti-GU efficacy of pharmaceuticals, and was used in this study. As shown in Figure 2A, a concise graphic scheme was drafted to show the essential procedures and animal grouping. At the end of the experiment, gastric tissues from all rats were harvested/stained to assess structural changes or observe the production/expression levels of key molecules.

**FIGURE 3:**
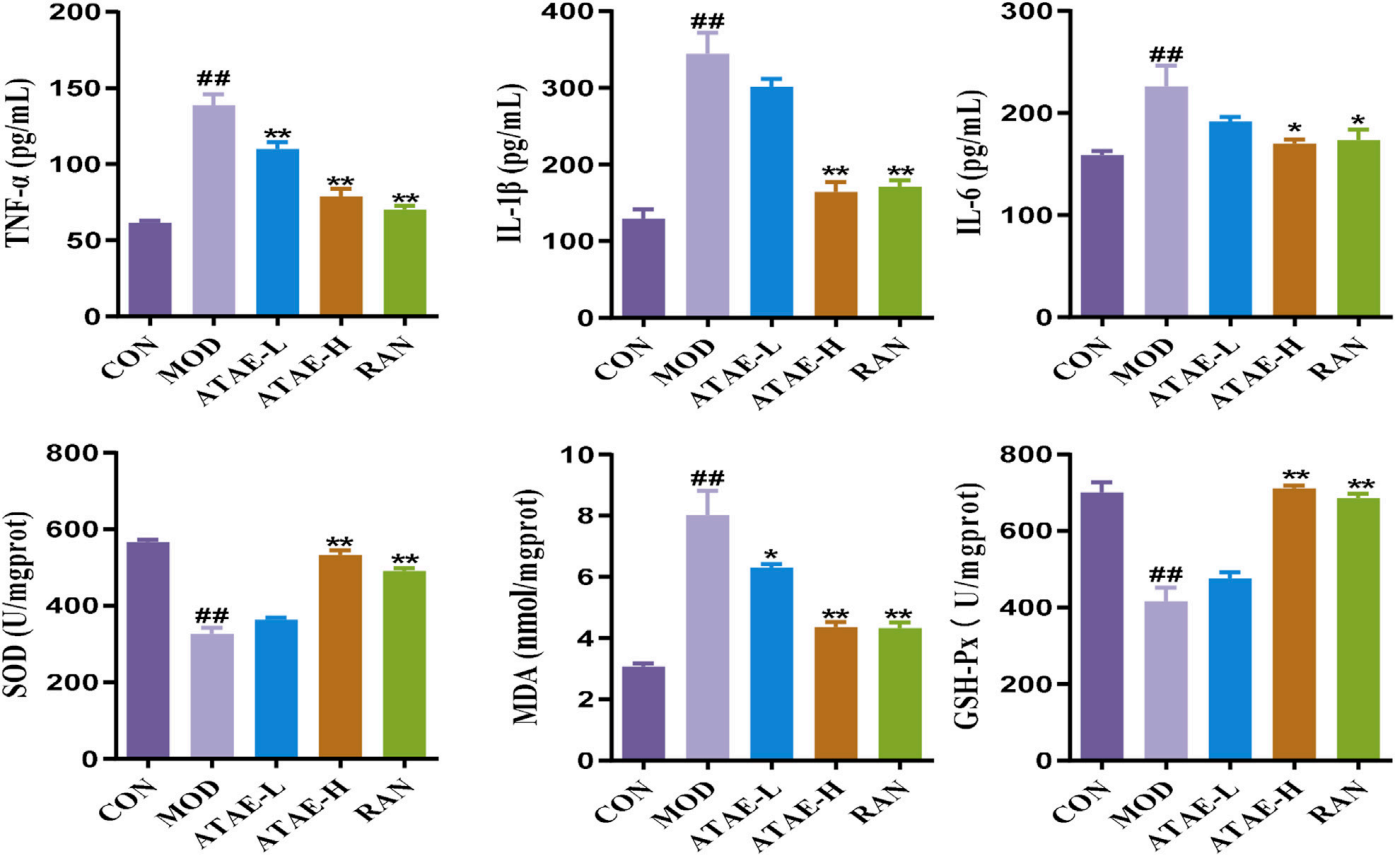
ATAE modulated the levels of TNF-a, IL-1β, IL-6, SOD, GSH-Px, and MDA in gastric tissues (n = 8). Data are expressed as mean ± SEM. ##*p* < 0.01 vs CON group; *p < 0.05 and **p < 0.01 vs the MOD group.

As the tissue morphology indicated (the upper panel of Figure 2B and the left panel of Figure 2C), ATAE treatment significantly reduced the visible damage to gastric tissue, characterized by a decrease in hemorrhagic lesions and submucosal edema compared to the MOD group. As revealed in the middle panels of Figures 2B,C, compared with the MOD group, the ATAE-treated group displayed a substantial improvement in gastric mucosal damage and a significant reduction in histopathological scores. As the PAS staining results indicated (the lower panel of Figure 2B and the right panel of Figure 2C **)**, compared to the MOD group, a darker magenta color (indicating more glycoprotein content) was observed in the ATAE-treated groups, suggesting ATAE’s protective effects on the mucus layer. Mucus serves as a cytoprotective barrier by sequestering bicarbonate and neutralizing acid, along with protecting epithelial cells from luminal irritants. PAS staining is specifically and widely used to detect glycoproteins such as mucins, which are essential constituents of the mucus layer (Allen and Flemström, 2005; Sheng and Hasnain, 2022). The PAS staining results directly revealed that the gastric mucosa of ATAE-treated rats retained the mucus content (stained magenta), in contrast to that of the MOD group. The preservation of mucus in the ATAE groups correlated with reduced ulcer scores, indicating that ATAE could attenuate HCl/EtOH-induced mucus depletion, a characteristic feature of mucosal injury. Pro-inflammatory cytokine mediators including TNF-α, IL-1β, and IL-6 contribute to gastric ulceration by promoting inflammation and tissue damage, and oxidant/anti-oxidant mediators such as SOD, MDA, and GSH-Px play a role in ulcer development by causing oxidative stress and impairing mucosal defense mechanisms. Enhancing SOD, CAT, and GSH-Px activities or reducing MDA levels could protect the gastric mucosa, suggesting antioxidant therapy as a potential ulcer treatment strategy (Akmal et al., 2023; Liu et al., 2017). GU involves oxidative stress, where ROS damage gastric mucosa. SOD converts superoxide radicals (O_2_^−^) to H_2_O_2_. CAT and GSH-Px further break down H_2_O_2_ into water, preventing ROS accumulation. Reduced activity of these enzymes exacerbates oxidative injury. MDA, a lipid peroxidation marker, reflects ROS-induced damage. The measurement of the above-mentioned mediators in rat gastric tissues is shown in Figure 3. As indicated, the modeling-caused increases in concentrations of mediators, including TNF-α, IL-1β, IL-6, and MDA, in rat gastric tissues were significantly inhibited by ATAE treatment, while the reductions in concentrations of anti-oxidant SOD and GSH-Px in the MOD group were markedly restored after ATAE treatment. These results demonstrated that ATAE could notably ameliorate HCl/EtOH-induced GU in rats and exert potent anti-inflammatory and anti-oxidant activities.

### 3.3 Bioinformatics-guided mechanistic exploration of ATAE in treating GU

**FIGURE 4:**
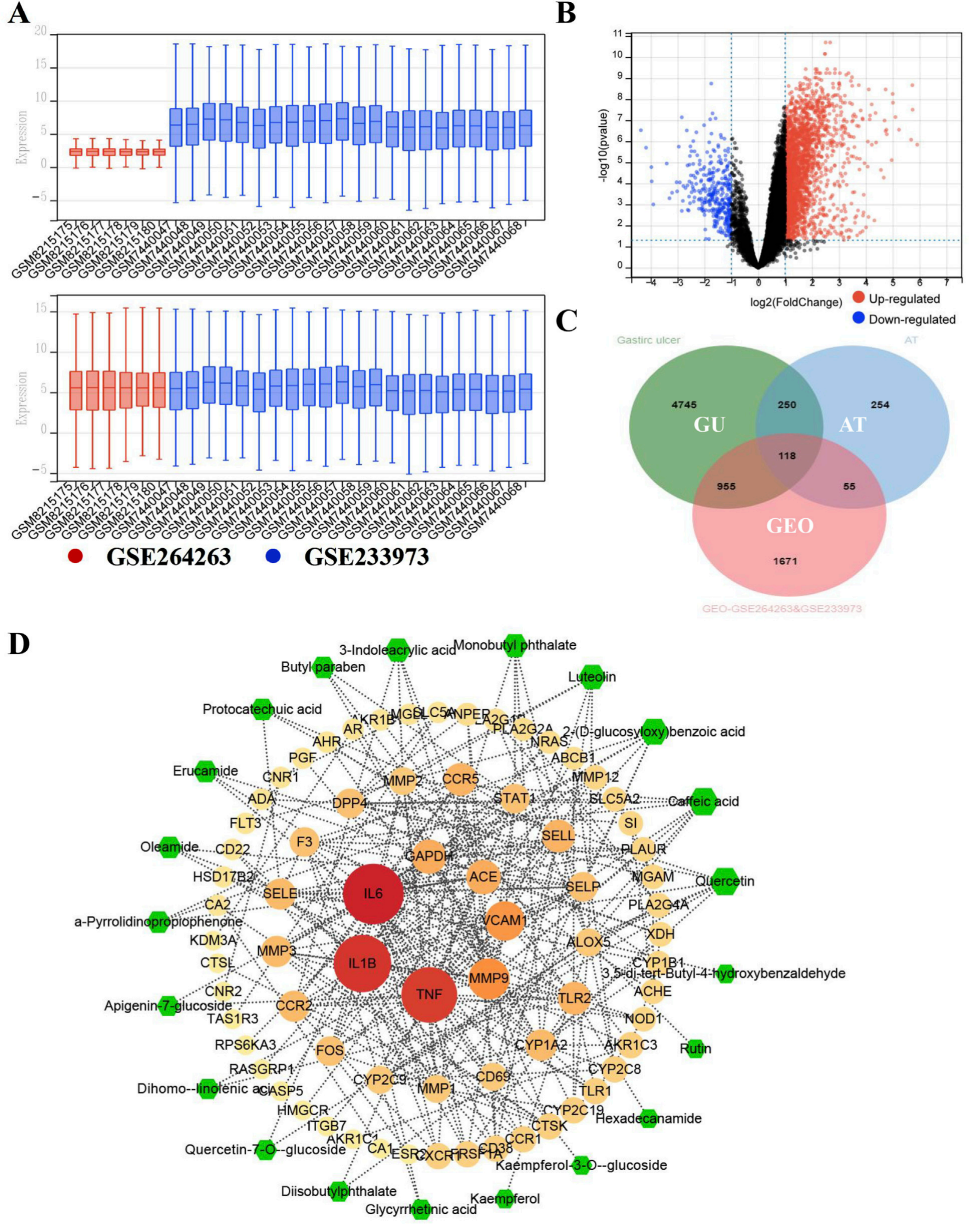
Bioinformatics-guided exploration on hub genes and key effective substances in ATAE against GU. **(A)** The normalization of GEO datasets GSE264263 and GSE233973. **(B)** Volcano diagram for the gene distribution. **(C)** Venn diagram for common targets among data clusters. **(D)** Network for key effective substances in ATAE and key GU-related target genes.

To explore/ensure the involved hub genes/pathway in the anti-GU effects of ATAE, the GEO data in this bioinformatics study were first normalized and visualized using boxplots (Figure 4A)**.** As shown, the medians and interquartile ranges (IQRs) of different datasets are more aligned and comparable after normalization, indicating that the variations between groups are now more reflective of true biological differences rather than technical variations. DEGs were then plotted on a volcano plot (Figure 4B). As indicated, a total of 2,799 DGEs were identified, with upregulated genes highlighted in red and downregulated genes in blue. In addition, we integrated the GEO DEGs, the GU targets (sourced from databases including GeneCards, OMIM, and TTD), and genes targeted by substances (i.e., analysts identified in ATAE using UPLC-MS). As shown in the Venn diagram (Figure 4C), a total of 118 overlapped genes form three clusters were identified and then uploaded to the STRING database to construct a core PPI network, indicating 60 nodes (Figure 4D). The node size and color (from pale yellow to red) were both proportional to the corresponding degree value. A total of 24 nodes/genes with greater degree values (>5, average degree value of all nods in the network) are shown in the innermost two layers and considered hub genes, and others are shown in the outermost layer. To indicate the effective substances in the network, the sizes of green hexagons shown in the outermost layer were proportional to the degree value based on substance–target (the aforementioned 24 hub genes) interactions and then merged into the above PPI network. Substances of quercetin and caffeic acid, possessing anti-GU effects as previous studies reported (Alkushi and Elsawy, 2017; Tian et al., 2014), are shown as green hexagons in the equal and largest size, probably contributing greatly to the efficacy of ATAE in treating GU.

**FIGURE 5:**
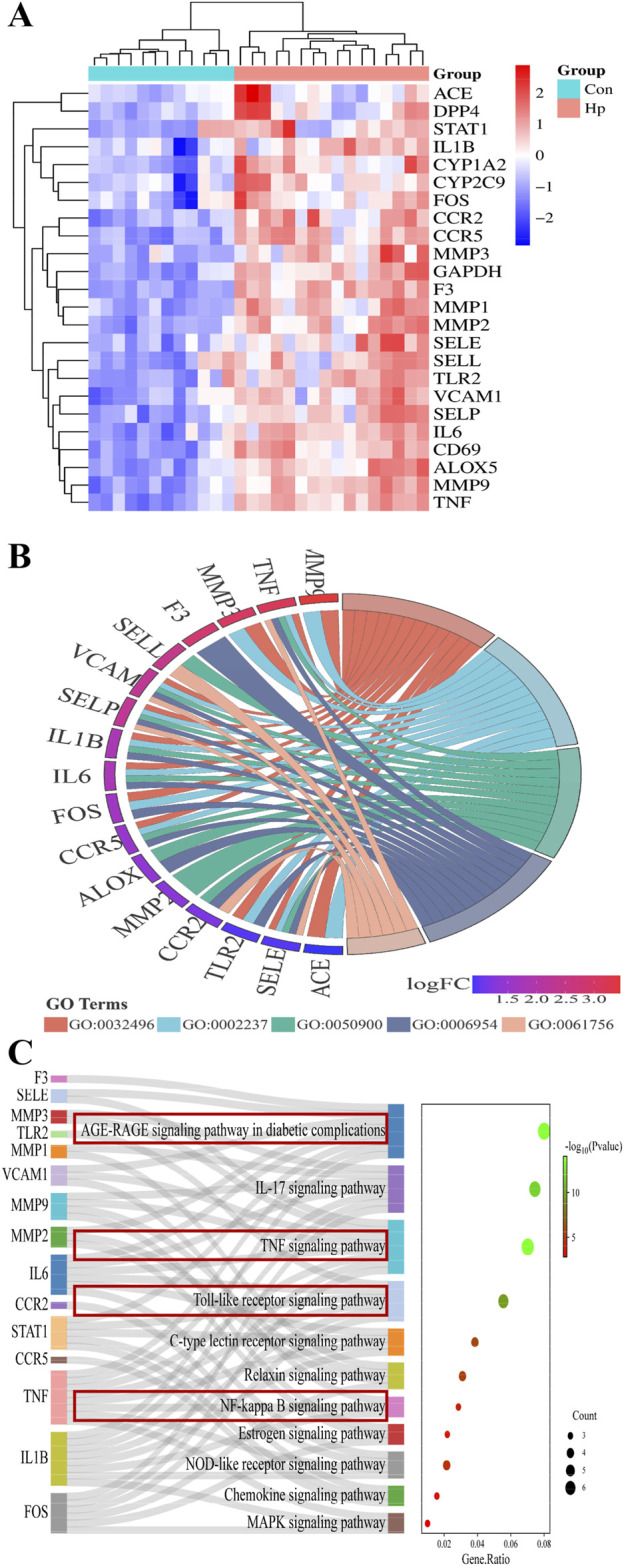
GO and KEGG pathway enrichment analyses of hub genes. **(A)** Heatmaps for the expression patterns of the 24 identified hub genes. **(B)** The gene ontology (GO) chord plot of top five ranked overrepresented GO terms belonging to the biological process (GO:0032496, response to lipopolysaccharide; GO:0002237, response to the molecule of bacterial origin; GO:0050900, leukocyte migration; GO:0006954, inflammatory response; GO:0061756, leukocyte adhesion to vascular endothelial cell). **(C)** The Sankey diagram for the KEGG pathway analysis.

As the heatmap of 24 hub genes (Figure 5A) suggested, all hub genes exhibited high expression levels in the HP-infected samples and low expression levels in the normal samples. As the GO term enrichment analyses indicated (Figure 5B **)**, 24 hub genes were enriched in the five most significant GO terms belonging to the biological processes: response to LPS, response to the molecule of bacterial origin, leukocyte migration, inflammatory response, and leukocyte adhesion to vascular endothelial cell. Of interest, these GO terms are well-implicated in the previously reported pathological studies on GU. Specifically, GU results from the imbalance between aggressive factors and mucosal defense. HP’s LPS initiates an inflammatory response by triggering leukocyte migration and adhesion to endothelial cells, which then release cytokines to combat infection and promote ulcer healing (FitzGerald and Smith, 2021). Furthermore, as the KEGG enrichment analyses indicated (Figure 5C **)**, the enriched key molecules/signaling pathways included AGE-RAGE, IL-17, TNF-a, Toll-like receptor (TLR), NF-κB, and NOD-like receptor (NLR). As numerous studies demonstrated, TLRs, TNFR (TNF-a’s receptor), and RAGE play a pivotal role in innate immunity activated by oxidative stress and inflammatory stimuli like LPS. Upon activation, they initiate signaling cascades that lead to the production of ROS subsequently activating Akt/NF-κB signaling and triggering inflammatory responses (Kawai et al., 2024; Li and Wu, 2021). Collectively, these *in silico* results highlighted the interrelated nature of oxidative stress and inflammatory responses in the pathogenesis of GU, and the molecular mechanism underlying ATAE’s anti-GU effects probably involves inhibition of the ROS/Akt/NF-κB signaling pathway.

### 3.4 ATAE inhibited the phosphorylation of Akt, IκBα, and NF-κB p65 in gastric tissues of GU rats

**FIGURE 6:**
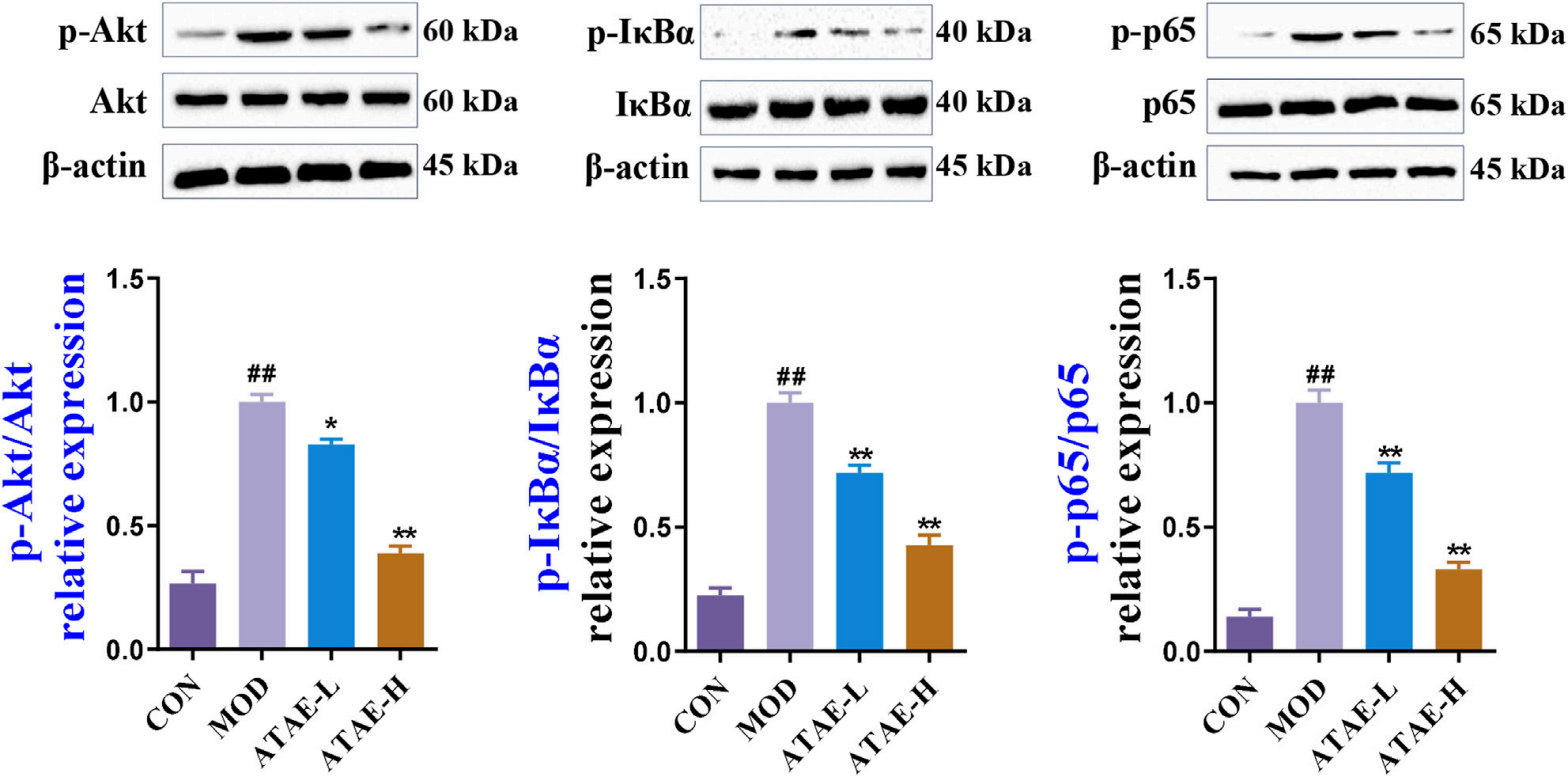
ATAE inhibited the phosphorylation of Akt, IκBα, and NF-κB p65 in gastric tissues of GU rats. Representative immunoblotting results of phosphorylated and total protein levels of Akt, IκBα, and NF-κB p65 in gastric tissues of GU rats (upper panel). Densitometric values are normalized to those in the MOD group and presented as the relative intensity (lower panel, n = 3). Data are expressed as mean ± SEM. ##*p* < 0.01 vs CON group; *p < 0.05 and **p < 0.01 vs MOD group.

To determine whether the Akt/NF-κB signaling pathway is involved in the anti-GU effects of ATAE, we determined the protein levels of phosphorylated and nonphosphorylated forms of Akt, IκBα, and NF-κB p65 in the rat gastric tissues. As shown by immunoblotting data in Figure 6 ATAE treatment did not alter the total protein levels of Akt, IκBα, and NF-κB p65; however, it notably reduced the levels of their phosphorylated forms in the GU tissues in a dose-dependent manner.

IκBα phosphorylation is a critical event in NF-κB activation. As observed in numerous pharmacological studies, the ratio of phosphorylated IκBα to total IκBα is extensively utilized and accepted as a crucial indicator of NF-κB activation, without the additional evaluation on the total protein levels of IκBα alone (Liao et al., 2024; Shen et al., 2022; Wang M. M. et al., 2024). Nevertheless, it is important to note that alterations in the total protein level of IκBα can be triggered by complex mechanisms involving alternative phosphorylation sites or interactions with other cellular factors (Li et al., 2024; Wang W. T. et al., 2024). Overall, our immunoblotting results are absolutely in agreement with observations in many previously reported GU-associated studies (Gong et al., 2022; Hossen et al., 2019; Li et al., 2023), suggesting the involvement of the Akt/NF-κB signaling pathway in the anti-GU effects of ATAE.

### 3.5 ATAE modulated the levels of ROS/Akt/NF-κB-mediated cytokines/molecules

To follow/validate the findings of the bioinformatics study, although GES-1 cells, originating from gastric epithelium, offer a direct model for gastric disease research due to their tissue-specific relevance, RAW264.7 cells have gained broader application in the study of inflammation and oxidative stress mechanisms (Li et al., 2023). They are broadly recognized and utilized for their responsiveness to various stimuli, thereby making them ideal for modeling inflammation/oxidant-driven gastric pathologies (Gu et al., 2023; Wang et al., 2023). Accordingly, LPS-induced RAW264.7 cells were used in this study to explore the anti-inflammatory and antioxidant properties of ATAE and underlying mechanism.

**FIGURE 7:**
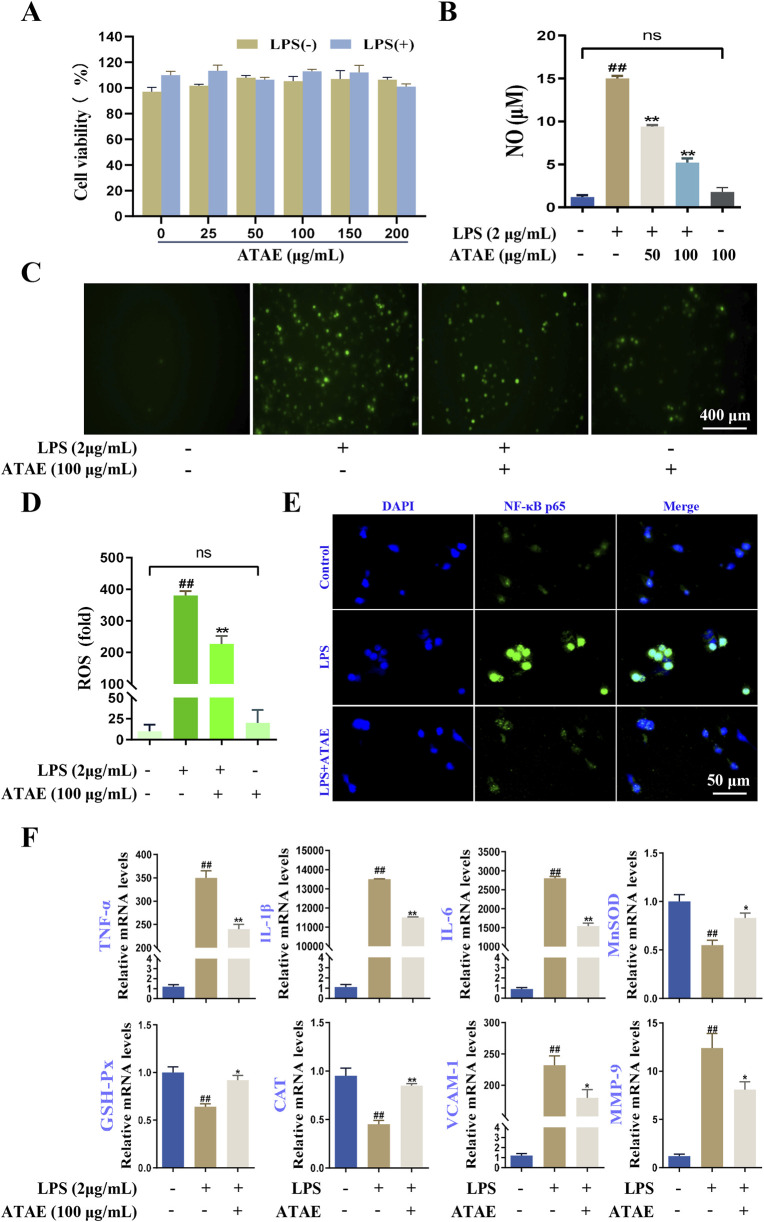
ATAE diminished ROS production, NF-κB p65 nuclear localization, and mRNA expression levels of multiple mediators regulated by NF-κB signaling in LPS-stimulated RAW264.7 cells. **(A)** Effects of ATAE on the cell viability in the absence or presence of LPS for 24 h. **(B)** Inhibitory effects of ATAE on NO productions. **(C)** Fluorescence microscopy imaging of ROS productions (scale bar: 400 μm). **(D)** Statistical analyses on ROS fluorescence intensities in **C**. **(E)** Nuclear localization of NF-κB p65 (scale bar: 50 μm). **(F)** mRNA expression levels of TNF-a, IL-1β, IL-6, MnSOD, GSH-Px, MDA, VCAM-1, and MMP-9. For **C–F**, the concentrations of ATAE and LPS used were 100 and 2 μg/mL, respectively. Data are expressed as mean ± SEM (n = 3). ##*p* < 0.01 vs control group; *p < 0.05 and **p < 0.01 vs group treated with LPS individually.

MTT assays were performed to assess the effects of ATAE on RAW264.7 cell viability under conditions with or without 2 μg/mL LPS. After a 24-h incubation, cell viability remained above 90% even with concentrations of ATAE up to 200 μg/mL (Figure 7A). Then, ATAE at concentrations of 50 and 100 μg/mL was used in subsequent assays. As indicated, NO production in the LPS-stimulated cells was notably and dose-dependently decreased by ATAE (Figure 7B). Additionally, NO production in cells individually treated with ATAE (100 μg/mL) was not altered, compared with that in normal cells. Likewise, upregulated levels of ROS in LPS-induced cells were obviously decreased by ATAE; relative to normal cells, no change in ROS levels was observed after ATAE treatment individually (Figures 7C,D). These results demonstrated the potent inhibitory effects of ATAE on the essential inflammatory and oxidant mediators in modeling cells, with no irritation observed in normal cells. As reported, ROS can activate the Akt pathway, leading to the phosphorylation and degradation of IκBα, which allows NF-κB to translocate to the nucleus and induce the expression of pro-inflammatory genes (Xie et al., 2022; Xu et al., 2023). As shown in the immunofluorescence assay (Figure 7E), ATAE treatment markedly reduced the nuclear translocation of NF-κB p65 in LPS-stimulated cells, under conditions similar to those reported in previous studies (Choi et al., 2018; Kim et al., 2021). Furthermore, the mRNA expression levels of NF-κB-regulated genes of the six above-determined mediators (TNF-α, IL-1β, IL-6, MnSOD, GSH-Px, and CAT) and two additional inflammation-associated hub genes (VCAM-1 and MMP-9) obtained in bioinfomatics study were notably modulated by ATAE in modeling cells (Figure 7F). These findings suggested the modulating effects of ATAE on the expression levels of ROS, NF-κB nuclear translocation, and NF-κB-regulated pro-inflammatory mediators, again demonstrating that inhibition of the ROS/Akt/NF-κB signaling pathway is involved in the GU-ameliorating effects of ATAE.

## 4 Discussion

Due to the side effects of current anti-GU medications, pharmaceutical and medical scientists are exploring safer and effective alternatives from medicinal plants or TCM formulas (Bi et al., 2014; Gong et al., 2024). As mentioned earlier, the herbal medicine AT in TCM clinics is primarily used to treat gastrointestinal diseases, such as dysentery (NJUCM, 2014). Despite distinct symptoms and pathogenic mechanisms, dysentery and GU are both inflammatory disorders of the digestive tract (Gong et al., 2024; Kataria et al., 2024; Williams and Berkley, 2018). As described in the TCM theory, AT works in “Spleen and Stomach Meridians” (*Piwei Jing* in Chinese), suggesting the potentials for treating/benefiting digestive system disorders. Currently, there is no evidence of significant toxicity of AT in animals. In fact, extracts from its leaves have been tested in mice and demonstrated protective rather than toxic effects in a model of acute lung injury (Tian et al., 2019). This indicates that, at least at the tested doses, AT does not exhibit notable toxicity in animals and may even possess therapeutic potential. However, further long-term or acute toxicity studies are needed to fully determine its safety profile. Furthermore, flavonoids and organic acids are abundant in AT, which are well known for their significant anti-inflammatory and antioxidant properties. As widely demonstrated, the interplay between anti-inflammatory and antioxidant properties plays a crucial role in ulcer pathogenesis and healing. Effective anti-ulcer therapies could ideally target both mechanisms (Bhol et al., 2024; Hu et al., 2021). Taken together, these inspired us to ascertain whether this herb could be used as a potential anti-ulcer agent for treating digestive disorders like GU. To explore this notion, we investigated the anti-ulcer activities of AT and underlying mechanisms by using pharmacological and bioinformatics approaches.

For pharmacological investigation, GU modeling is essential for studying ulcer pathogenesis and potential treatments. Animal models for GU typically include chemical induction methods, such as the administration of non-steroidal anti-inflammatory drugs (NSAIDs) or ethanol, and surgical methods, such as pyloric ligation (Gong et al., 2024). These models are favored because of controlled experimentation and reproducibility. The HCl-/EtOH-induced GU model is widely used due to its simplicity and effectiveness. In this model, animals are typically fasted for 24 h before being administered with a combination of HCl and ethanol. The HCl lowers the pH in the stomach, creating an acidic environment that weakens the gastric mucosal barrier. Ethanol then further disrupts the mucosal lining, leading to oxidative stress and inflammation, ultimately causing acute gastric mucosal damage and ulcer formation (Cho et al., 2023; Wang et al., 2021). Additionally, the model is easy to replicate and yields consistent results, making it suitable for screening anti-ulcer agents. As we demonstrated, ATAE could notably ameliorate HCl/EtOH-induced GU in rats and exert potent anti-inflammatory and anti-oxidant activities, providing robust evidence for the use of AT in treating GU.

GU can be caused by multiple factors, such as bacterial infection, dietary/lifestyle habits (e.g., long-term alcohol consumption, irregular diet, and smoking), psychological stress, and genetic predisposition. Among these, HP plays a significant role in the development of GU (FitzGerald and Smith, 2021; Jin et al., 2024). Although HP is acknowledged as the most frequent etiological factor in the progression of GU (Malfertheiner et al., 2023), the infection does not uniformly lead to ulceration in all individuals, accordingly not being prevalent for GU modeling in animals (Burkitt et al., 2017). Furthermore, although certain models in mice, Mongolian gerbils, and non-human primates have shown promise in establishing HP infection, their widespread application still faces multiple challenges. These challenges include significant species-specific differences, variable reproducibility, technical complexity, ethical considerations, and cost constraints (Ansari and Yamaoka, 2022; Liu et al., 2024). Given the importance of HP in GU, it is fascinating and insightful to explore the key molecules or signaling pathways based on bioinformatics alterations in HP-infected biospecimens.

**FIGURE 8:**
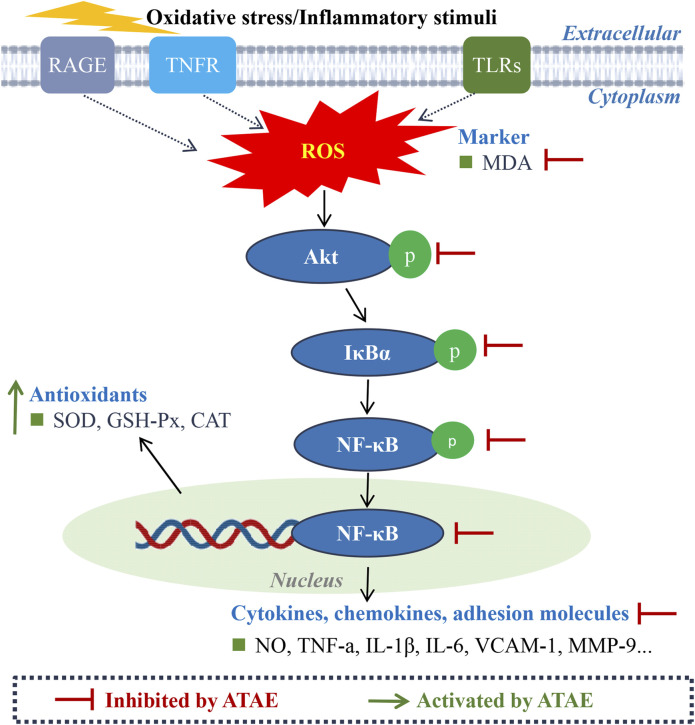
Schematic diagram of the molecular mechanism underlying the anti-GU effects of ATAE.

For bioinformatics investigation, as illustrated in Figure 8, our pathway enrichment analyses indicated that HP infection could be closely related to the activation of RAGE, TNFR, TLR, and NF-κB signaling. As reported, these pathways are intricately involved in the regulation of oxidative stress and inflammatory responses. ROS is crucial in the development of gastritis and GU, acting as a hub that integrates signals from various upstream receptors, such as RAGE, TNF, and TLRs. As described in numerous studies (Badr et al., 2019; Krajka-Kuzniak and Baer-Dubowska, 2021), these receptors, when activated, can lead to the production of ROS, which, in turn, activates key proteins including Akt and IκBα in the NF-κB inflammatory pathway. The interplay among these elements orchestrates a cascade of events leading to cellular dysfunction and tissue damage in GU models. Furthermore, our experimental results further corroborated the involvement of these key events in the drug’s efficacy. As indicated, ATAE significantly modulated the generation of ROS and inhibited the activation of Akt, IκBα, and NF-κB p65. Moreover, it effectively suppressed the production or mRNA expression levels of NF-κB-mediated inflammatory mediators, thereby attenuating oxidative stress and inflammation in the gastric mucosa. These regulatory effects of ATAE on the key molecules and mediators of ROS/Akt/NF-κB signaling pathways underscore the molecular mechanism involved in the herb’s efficacy against experimental GU.

The canonical NF-κB pathway (p50/p65) mediates acute inflammation through rapid activation of TNF-α, IL-6, and IL-1β, which exacerbates mucosal damage by increasing oxidative stress (reduced SOD/GSH-Px and elevated MDA). Acting as the rate-limiting step, it initiates the inflammatory cascade (Liu et al., 2017; Mitchell and Carmody, 2018; Rius-Perez et al., 2020). In contrast, the non-canonical pathway (p52/RelB) orchestrates chronic inflammation and tissue remodeling by sustaining VCAM-1-mediated leukocyte adhesion and MMP-9-dependent extracellular matrix degradation. These pathways exhibit significant crosstalk: canonical responses drive initial ulcer formation, while non-canonical signaling activation perpetuates tissue injury and impairs healing injury (Mukherjee et al., 2024; Sun, 2017). Dual-pathway targeting may offer comprehensive ulcer therapy by addressing both inflammatory and remodeling phases. Additionally, the crosstalk between the Nrf2 and NF-κB pathways is complex and bidirectional and could mediate critical inflammatory and oxidative events in experimental GU (Badr et al., 2019). Activation of NF-κB can suppress Nrf2 activity. Conversely, when activated, Nrf2 first dissociates from its repressor Kelch-like ECH associating protein 1 (Keap1) in the cytosol and then translocates to the nucleus, where it upregulates the expressions of genes such as SOD and GSH-Px; the activated Nrf2 can negatively regulate NF-κB signaling by decreasing intracellular ROS levels, ultimately inhibiting NF-κB nuclear translocation. As shown in our additional findings, ATAE treatment significantly increased the mRNA level of Nrf2 while notably decreasing the mRNA level of Keap1 in modeling cells (Supplementary Figure S2). These regulatory effects are consistent with those of previously reported studies (Fan et al., 2018; Liu et al., 2022), suggesting the activating effects of ATAE on Nrf2 signaling. Collectively, these results demonstrated the involvement of modulating crosstalk of Nrf2 and NF-κB signaling in the drug’s anti-GU effects, thus deserving further study. Numerous studies have reported the synergism of phytochemicals in modulating these two signaling pathways (Krajka-Kuzniak and Baer-Dubowska, 2021; Shah et al., 2024). Given this, it is probably an effective strategy and warranted in our subsequent study to uncover the effective combination of phytochemicals that could both act on Nrf2 and NF-κB, providing novel scientific evidence and additional alternative therapy for the use of AT for treating GU. In addition, although bioinformatics offers an immense value in drug discovery and mechanistic elucidation, it is an inevitable challenge for data quality, computational complexity, and the dynamic nature. Hence, investigations using multi-omics approaches (e.g., proteomics and metabolomics) (Liu et al., 2023; Zhang et al., 2024) are warranted in our future study to provide a more comprehensive understanding of the mechanism of action of AT against GU.

In summary, this work provides robust evidence on elucidating the effects and mechanism of action of ATAE in treating experimental GU by using bioinformatic and pharmacological approaches. The findings highlight the involvement of the ROS/Akt/NF-κB signaling pathway in ATAE’s efficacy against GU by mitigating oxidative stress and inflammatory responses. Further exploration of the clinical potential of ATAE and optimization of its therapeutic application are warranted in future research.

## 5 Conclusion

ATAE ameliorates oxidative stress and inflammatory responses against HCl/EtOH-induced GU in rats, which are probably associated with inhibiting the ROS/Akt/NF-κB signaling pathway. The novel findings of this study, for the first time, provide scientific justifications for the use of AT in treating peptic ulcer. Future studies are warranted to elucidate the clinical potential and a more comprehensive understanding of underlying mechanisms of AT in treating GU.

## Data Availability

The raw data supporting the conclusions of this article will be made available by the authors, without undue reservation.

## Supplementary material

Supplementary Figure S1 Pilot study: ATAE protects against experimental GU in rats. Rats were randomly divided into two groups (n = 3/group): model (MOD) group and ATAE at the dosage of 100 mg/kg (ATAE-H) group. Rats in the MOD group were i.g. dosed with 1% CMC-Na (vehicle), and rats in the ATAE-H group were i.g. dosed with vehicle containing 100 mg/kg of ATAE, twice daily for 3 consecutive days. On day 4, following a 30-min fast after the last dosing, each rat was i.g. dosed with 10 mL/kg of the hydrochloric acid/ethanol (HCl/EtOH) solution. All rats were further fasted for 1 h and then euthanized by the inhalation of carbon dioxide. Gastric tissues were collected. The ulcer index is shown. Data are presented as mean ± SD (n = 3).

Supplementary Figure S2 ATAE markedly modulated mRNA expression levels of Nrf2 and Keap1 in LPS-stimulated RAW264.7 cells. The concentrations of ATAE and LPS used were 100 and 2 μg/mL, respectively. Data are expressed as mean ± SEM (n = 3). **p* < 0.05 and ***p* < 0.01 vs group treated with LPS individually.

## Abbreviations

AT, *Abutilon theophrasti* Medic.; ATAE, aqueous extract of AT; GU, gastric ulcer; HCl/EtOH, hydrochloric acid/ethanol; GI, gastrointestinal; GEO, Gene Expression Omnibus; DGE, differentially expressed gene; HP, *Helicobacter pylori*; H&E, hematoxylin and eosin; PAS, periodic acid–Schiff; LPS, lipopolysaccharide; ROS, reactive oxygen species; NO, nitric oxide; SOD, superoxide dismutase; MDA, malondialdehyde; GSH-Px, glutathione peroxidase; CAT, catalase.

## References

[B1] Akash S. R. Tabassum A. Aditee L. M. Rahman A. Hossain M. I. Hannan M. A. (2024). Pharmacological insight of rutin as a potential candidate against peptic ulcer. Biomed. Pharmacother. 177, 116961. 10.1016/j.biopha.2024.116961 38901206

[B2] Akmal M. N. Aziz I. A. Azlina M. F. N. (2023). Piper sarmentosum Roxb. methanolic extract prevents stress-induced gastric ulcer by modulating oxidative stress and inflammation. Front. Pharmacol. 13, 971443. 10.3389/Fphar.2022.971443 36712695 PMC9879357

[B3] Alameer A. Chicco D. (2022). geoCancerPrognosticDatasetsRetriever: a bioinformatics tool to easily identify cancer prognostic datasets on Gene Expression Omnibus (GEO). Bioinformatics 38 (6), 1761–1763. 10.1093/bioinformatics/btab852 34935889

[B4] Alkushi A. G. R. Elsawy N. A. M. (2017). Quercetin attenuates, indomethacin-induced acute gastric ulcer in rats. Folia Morphol. (Warsz.) 76 (2), 252–261. 10.5603/FM.a2016.0067 27813628

[B5] Allen A. Flemström G. (2005). Gastroduodenal mucus bicarbonate barrier: protection against acid and pepsin. Am. J. Physiol. Cell Physiol. 288 (1), C1–C19. 10.1152/ajpcell.00102.2004 15591243

[B6] Ansari S. Yamaoka Y. (2022). Animal models and *Helicobacter pylori* infection. J. Clin. Med. 11 (11), 3141. 10.3390/jcm11113141 35683528 PMC9181647

[B7] Badr A. M. El-Orabi N. F. Ali R. A. (2019). The implication of the crosstalk of Nrf2 with NOXs, and HMGB1 in ethanol-induced gastric ulcer: potential protective effect is afforded by Raspberry Ketone. PLoS One 14 (8), e0220548. 10.1371/journal.pone.0220548 31404064 PMC6690542

[B8] Bhol N. K. Bhanjadeo M. M. Singh A. K. Dash U. C. Ojha R. R. Majhi S. (2024). The interplay between cytokines, inflammation, and antioxidants: mechanistic insights and therapeutic potentials of various antioxidants and anti-cytokine compounds. Biomed. Pharmacother. 178, 117177. 10.1016/j.biopha.2024.117177 39053423

[B9] Bi W. P. Man H. B. Man M. Q. (2014). Efficacy and safety of herbal medicines in treating gastric ulcer: a review. World J. Gastroenterol. 20 (45), 17020–17028. 10.3748/wjg.v20.i45.17020 25493014 PMC4258570

[B10] Burkitt M. D. Duckworth C. A. Williams J. M. Pritchard D. M. (2017). Helicobacter pylori-induced gastric pathology: insights from *in vivo* and *ex vivo* models. Dis. Model. Mech. 10 (2), 89–104. 10.1242/dmm.027649 28151409 PMC5312008

[B11] Chen W. Deng Y. Y. Yu J. W. Leung Y. T. Bai J. X. Chen Y. J. (2023). A tri-herb formulation protects against ethanol-induced mouse liver injury and downregulates mitogen-activated protein kinase phosphatase 1. Phytomedicine 114, 154802. 10.1016/j.phymed.2023.154802 37054486

[B12] Cho H. S. Kwon T. W. Kim J. H. Lee R. Bae C. S. Kim H. C. (2023). Gintonin alleviates HCl/ethanol- and indomethacin-induced gastric ulcers in mice. Int. J. Mol. Sci. 24 (23), 16721. 10.3390/ijms242316721 38069044 PMC10705886

[B13] Choi H. W. Shin P. G. Lee J. H. Choi W. S. Kang M. J. Kong W. S. (2018). Anti-inflammatory effect of lovastatin is mediated via the modulation of NF-κB and inhibition of HDAC1 and the PI3K/Akt/mTOR pathway in RAW264.7 macrophages. Int. J. Mol. Med. 41 (2), 1103–1109. 10.3892/ijmm.2017.3309 29207042

[B14] Fan M. Li Y. Yao C. Liu X. Liu J. Yu B. (2018). DC32, a dihydroartemisinin derivative, ameliorates collagen-induced arthritis through an nrf2-p62-keap1 feedback loop. Front. Immunol. 9, 2762. 10.3389/fimmu.2018.02762 30538709 PMC6277526

[B15] Feng L. Bao T. Bai L. Mu X. Ta N. Bao M. (2023). Mongolian medicine formulae Ruda-6 alleviates indomethacin-induced gastric ulcer by regulating gut microbiome and serum metabolomics in rats. J. Ethnopharmacol. 314, 116545. 10.1016/j.jep.2023.116545 37196816

[B16] FitzGerald R. Smith S. M. (2021). An overview of *Helicobacter pylori* infection. Methods Mol. Biol. 2283, 1–14. 10.1007/978-1-0716-1302-3_1 33765303

[B17] Gerlier D. Thomasset N. (1986). Use of MTT colorimetric assay to measure cell activation. J. Immunol. Methods 94 (1-2), 57–63. 10.1016/0022-1759(86)90215-2 3782817

[B18] Gong H. Zhao N. Zhu C. Luo L. Liu S. (2024). Treatment of gastric ulcer, traditional Chinese medicine may be a better choice. J. Ethnopharmacol. 324, 117793. 10.1016/j.jep.2024.117793 38278376

[B19] Gong M. Li Q. F. Guo H. Cui B. D. Liu Y. L. Wang P. (2022). Protective effect of active components of Eucommia ulmoides leaves on gastric ulcers in rats: involvement of the PI3K/Akt/NF-κB pathway. J. Food Sci. 87 (7), 3207–3222. 10.1111/1750-3841.16214 35733355

[B20] Gu T. Zhang Z. Liu J. Chen L. Tian Y. Xu W. (2023). Chlorogenic acid alleviates LPS-induced inflammation and oxidative stress by modulating CD36/AMPK/PGC-1α in RAW264.7 macrophages. Int. J. Mol. Sci. 24 (17), 13516. 10.3390/ijms241713516 37686324 PMC10487601

[B21] Guo H. Cui B. D. Gong M. Li Q. X. Zhang L. X. Chen J. L. (2024). An ethanolic extract of Arctium lappa L. leaves ameliorates experimental atherosclerosis by modulating lipid metabolism and inflammatory responses through PI3K/Akt and NF-κB singnaling pathways. J. Ethnopharmacol. 325, 117768. 10.1016/j.jep.2024.117768 38253275

[B22] Gupta A. Shetty S. Mutalik S. Chandrashekar H. R. K N. Mathew E. M. (2023). Treatment of *H. pylori* infection and gastric ulcer: need for novel Pharmaceutical formulation. Heliyon 9 (10), e20406. 10.1016/j.heliyon.2023.e20406 37810864 PMC10550623

[B23] Hossen M. J. Chou J. Y. Li S. M. Fu X. Q. Yin C. Guo H. (2019). An ethanol extract of the rhizome of Atractylodes chinensis exerts anti-gastritis activities and inhibits Akt/NF-κB signaling. J. Ethnopharmacol. 228, 18–25. 10.1016/j.jep.2018.09.015 30218812

[B24] Hu J. Luo J. Zhang M. Wu J. Zhang Y. Kong H. (2021). Protective effects of radix sophorae flavescentis carbonisata-based carbon dots against ethanol-induced acute gastric ulcer in rats: anti-inflammatory and antioxidant activities. Int. J. Nanomedicine 16, 2461–2475. 10.2147/IJN.S289515 33814910 PMC8009542

[B25] Jia Y. Wang M. Lambers P. H. van Andel T. (2024). The catalogue of the Westhoff collection of Chinese Materia Medica (c. 1870): evidence of interaction between a Chinese medicine practitioner and the Dutch in Indonesia. J. Ethnopharmacol. 318 (Pt B), 116987. 10.1016/j.jep.2023.116987 37531803

[B26] Jin L. X. Fang Y. P. Xia C. M. Cai T. W. Li Q. Q. Wang Y. Y. (2024). *Helicobacter pylori* infection alters gastric microbiota structure and biological functions in patients with gastric ulcer or duodenal ulcer. World J. Gastroenterol. 30 (24), 3076–3085. 10.3748/wjg.v30.i24.3076 38983956 PMC11230059

[B27] Karri R. L. Amrutha R. Shrinivas Bojji M. Kumar K. M. Benarji K. A. (2024). Analyzing pooled microarray gene expression data to uncover common pathways in periodontitis and oral squamous cell carcinoma from the gene expression omnibus. J. Pharm. Bioallied Sci. 16 (Suppl. 2), S1515–S1521. 10.4103/jpbs.jpbs_1180_23 38882729 PMC11174343

[B28] Kataria S. Singla A. Sharma C. (2024). Unveiling *Balantidium coli*: a rare protozoan causing a series of cases of dysentery in Rajasthan and review of literature. J. Postgrad. Med. 70 (4), 242–244. 10.4103/jpgm.jpgm_509_24 39660569 PMC11722708

[B29] Kawai T. Ikegawa M. Ori D. Akira S. (2024). Decoding Toll-like receptors: recent insights and perspectives in innate immunity. Immunity 57 (4), 649–673. 10.1016/j.immuni.2024.03.004 38599164

[B30] Kim M. Kim J. Y. Yang H. S. Choe J. S. Hwang I. G. (2021). Nepetoidin B from *Salvia plebeia* R. Br. inhibits inflammation by modulating the NF-κB and Nrf2/HO-1 signaling pathways in macrophage cells. Antioxidants (Basel) 10 (8), 1208. 10.3390/antiox10081208 34439456 PMC8388923

[B31] Krajka-Kuzniak V. Baer-Dubowska W. (2021). Modulation of Nrf2 and NF-kappaB signaling pathways by naturally occurring compounds in relation to cancer prevention and therapy. Are combinations better than single compounds? Int. J. Mol. Sci. 22 (15). 10.3390/ijms22158223 PMC8348704 34360990

[B32] Li D. Wu M. (2021). Pattern recognition receptors in health and diseases. Signal Transduct. Target. Ther. 6 (1), 291. 10.1038/s41392-021-00687-0 34344870 PMC8333067

[B33] Li Q. X. Guo H. Gong M. Zhang Y. Yang L. H. Wang J. (2023). Protective effects of aqueous extracts of the herb of paederia scandens (lour.) merr. Against HCl/EtOH-Induced gastric ulcer in rats: involvement and inhibitors’ identification of NF-κB signaling. J. Food Biochem. 2023, 1–15. 10.1155/2023/4545188

[B34] Li S. Z. Shu Q. P. Zhou H. M. Liu Y. Y. Fan M. Q. Liang X. Y. (2024). CLK2 mediates IκBα-independent early termination of NF-κB activation by inducing cytoplasmic redistribution and degradation. Nat. Commun. 15 (1), 3901. 10.1038/s41467-024-48288-z 38724505 PMC11082251

[B35] Liao J. Zhao W. Zhang Y. Zou Z. Zhang Q. Chen D. (2024). Dendrobium officinale Kimura et Migo polysaccharide ameliorated DNFB-induced atopic dermatitis in mice associated with suppressing MAPK/NF-κB/STAT3 signaling pathways. J. Ethnopharmacol. 335, 118677. 10.1016/j.jep.2024.118677 39121927

[B36] Liu J. Zhao L. Cai H. Zhao Z. Wu Y. Wen Z. (2022). Antioxidant and anti-inflammatory properties of rubber seed oil in lipopolysaccharide-induced RAW 267.4 macrophages. Nutrients 14 (7), 1349. 10.3390/nu14071349 35405962 PMC9003255

[B37] Liu T. Zhang L. Joo D. Sun S. C. (2017). NF-κB signaling in inflammation. Signal Transduct. Target. Ther. 2, 17023. 10.1038/sigtrans.2017.23 29158945 PMC5661633

[B38] Liu Y. Zhang X. Yang L. Zhou S. Li Y. Shen Y. (2023). Proteomics and transcriptomics explore the effect of mixture of herbal extract on diabetic wound healing process. Phytomedicine 116, 154892. 10.1016/j.phymed.2023.154892 37267693

[B39] Liu Z. Li H. Huang X. Liu Q. (2024). Animal models of *Helicobacter pylori* infection and vaccines: current status and future prospects. Helicobacter 29 (4), e13119. 10.1111/hel.13119 39108210

[B40] Maarouf A. Jones S. (2020). Lessons of the month: over-the-counter antacids causing hypercalcaemia: the emergence of calcium-alkali syndrome. Clin. Med.(Lond) 20 (4), e129–e130. 10.7861/clinmed.2020-0208 32675162 PMC7385791

[B41] Malfertheiner P. Camargo M. C. El-Omar E. Liou J. M. Peek R. Schulz C. (2023). *Helicobacter pylori* infection. Nat. Rev. Dis. Prim. 9 (1), 19. 10.1038/s41572-023-00431-8 37081005 PMC11558793

[B42] Mitchell J. P. Carmody R. J. (2018). NF-κB and the transcriptional control of inflammation. Int. Rev. Cell Mol. Biol. 335, 41–84. 10.1016/bs.ircmb.2017.07.007 29305014

[B43] Mukherjee T. Kumar N. Chawla M. Philpott D. J. Basak S. (2024). The NF-κB signaling system in the immunopathogenesis of inflammatory bowel disease. Sci. Signal. 17 (818), eadh1641. 10.1126/scisignal.adh1641 38194476

[B44] NJUCM (2014). Dictionary of Chinese Materia Medica, dictionary of Chinese Materia Medica. 2 ed. Shanghai: Shanghai Science and Technology Press, 1550.

[B45] Qiu X. Y. Yan L. S. Kang J. Y. Yu Gu C. Chi-Yan Cheng B. Wang Y. W. (2024). Eucalyptol, limonene and pinene enteric capsules attenuate airway inflammation and obstruction in lipopolysaccharide-induced chronic bronchitis rat model via TLR4 signaling inhibition. Int. Immunopharmacol. 129, 111571. 10.1016/j.intimp.2024.111571 38309095

[B46] Rius-Perez S. Perez S. Marti-Andres P. Monsalve M. Sastre J. (2020). Nuclear factor kappa B signaling complexes in acute inflammation. Antioxid. Redox Signal. 33 (3), 145–165. 10.1089/ars.2019.7975 31856585

[B47] Salem M. B. Saleh A. M. Seif El-Din S. H. Samir S. Hammam O. A. El-Lakkany N. M. (2024). Molecular docking, characterization, ADME/toxicity prediction, and anti-ulcer activity of new quercetin derivatives on indomethacin-induced gastric ulcer in mice. Toxicol. Appl. Pharmacol. 484, 116880. 10.1016/j.taap.2024.116880 38447874

[B48] Shah A. Varma M. Bhandari R. (2024). Exploring sulforaphane as neurotherapeutic: targeting Nrf2-Keap and Nf-Kb pathway crosstalk in ASD. Metab. Brain Dis. 39 (3), 373–385. 10.1007/s11011-023-01224-4 37249861

[B49] Shen Y. Teng L. Qu Y. Liu J. Zhu X. Chen S. (2022). Anti-proliferation and anti-inflammation effects of corilagin in rheumatoid arthritis by downregulating NF-κB and MAPK signaling pathways. J. Ethnopharmacol. 284, 114791. 10.1016/j.jep.2021.114791 34737112

[B50] Sheng Y. H. Hasnain S. Z. (2022). Mucus and mucins: the underappreciated host defence system. Front. Cell. Infect. Microbiol. 12, 856962. 10.3389/fcimb.2022.856962 35774401 PMC9238349

[B51] Sun S. C. (2017). The non-canonical NF-κB pathway in immunity and inflammation. Nat. Rev. Immunol. 17 (9), 545–558. 10.1038/nri.2017.52 28580957 PMC5753586

[B52] Tian C. Wang M. Liu X. Wang H. Zhao C. (2014). HPLC quantification of nine chemical constituents from the five parts of Abutilon theophrasti Medic. J. Chromatogr. Sci. 52 (3), 258–263. 10.1093/chromsci/bmt021 23580704

[B53] Tian C. Wang M. Shen C. Zhao C. (2012). Accuracy mass screening and identification of phenolic compounds from the five parts of *Abutilon theophrasti* Medic. by reverse-phase high-performance liquid chromatography-electrospray ionization-quadrupoles-time of flight-mass spectrometry. J. Sep. Sci. 35 (5-6), 763–772. 10.1002/jssc.201100775 22275244

[B54] Tian C. Zhang P. Yang C. Gao X. Wang H. Guo Y. (2018). Extraction process, component analysis, and *in vitro* antioxidant, antibacterial, and anti-inflammatory activities of total flavonoid extracts from *Abutilon theophrasti* Medic. Leaves. Mediat. Inflamm. 2018, 3508506. 10.1155/2018/3508506 PMC5872602 29725269

[B55] Tian C. Zhang P. Yang J. Zhang Z. Wang H. Guo Y. (2019). The protective effect of the flavonoid fraction of *Abutilon theophrasti* Medic. leaves on LPS-induced acute lung injury in mice via the NF-κB and MAPK signalling pathways. Biomed. Pharmacother. 109, 1024–1031. 10.1016/j.biopha.2018.10.197 30551352

[B56] Wang C. Jiang Z. Du M. Cong R. Wang W. Zhang T. (2024). Novel Ser74 of NF-κB/IκBα phosphorylated by MAPK/ERK regulates temperature adaptation in oysters. Cell Commun. Signal. 22 (1), 539. 10.1186/s12964-024-01923-0 39529137 PMC11552224

[B57] Wang M. M. Li Q. X. Ren B. J. Hao D. L. Guo H. Yang L. H. (2024). Ethanolic extract of *Arctium lappa* leaves alleviates cerebral ischemia reperfusion-induced inflammatory injury via HDAC9-mediated NF-κB pathway. Phytomedicine 129, 155599. 10.1016/J.Phymed.2024.155599 38669967

[B58] Wang R. Sun F. Ren C. Zhai L. Xiong R. Yang Y. (2021). Hunan insect tea polyphenols provide protection against gastric injury induced by HCl/ethanol through an antioxidant mechanism in mice. Food Funct. 12 (2), 747–760. 10.1039/d0fo02677h 33367402

[B59] Wang W. T. Zhang Y. Y. Li Z. R. Li J. M. Deng H. S. Li Y. Y. (2024). Syringic acid attenuates acute lung injury by modulating macrophage polarization in LPS-induced mice. Phytomedicine 129, 155591. 10.1016/j.phymed.2024.155591 38692075

[B60] Wang Y. Xiong Z. Li C. Liu D. Li X. Xu J. (2023). Multiple beneficial effects of aloesone from aloe vera on LPS-induced RAW264.7 cells, including the inhibition of oxidative stress, inflammation, M1 polarization, and apoptosis. Molecules 28 (4), 1617. 10.3390/molecules28041617 36838606 PMC9960963

[B61] Williams P. C. M. Berkley J. A. (2018). Guidelines for the treatment of dysentery (shigellosis): a systematic review of the evidence. Paediatr. Int. Child. Health 38 (Suppl. 1), S50–S65. 10.1080/20469047.2017.1409454 29790845 PMC6021764

[B62] Xie L. Y. Yang Z. Wang Y. Hu J. N. Lu Y. W. Zhang H. (2022). 1-*O*-Actylbritannilactone ameliorates alcohol-induced hepatotoxicity through regulation of ROS/Akt/NF-κB-Mediated apoptosis and inflammation. ACS Omega 7 (21), 18122–18130. 10.1021/acsomega.2c01681 35664604 PMC9161245

[B63] Xu Q. Cui F. Li X. Wang N. Gao Y. Yin S. (2023). Dangshen Huangjiu prevents gastric mucosal injury and inhibits Akt/NF-κB pathway. Food Funct. 14 (17), 7897–7911. 10.1039/d3fo00489a 37491882

[B64] Zhang W. J. Guo H. Li L. Y. Zhang M. M. Xu E. R. Dai L. P. (2024). Network pharmacology-based strategy integrated with molecular docking and *in vitro* experimental validation to explore the underlying mechanism of Fangji Huangqi decoction in treating rheumatoid arthritis. Acs Omega 9 (29), 31878–31889. 10.1021/acsomega.4c03495 39072058 PMC11270556

[B65] Zhang Z. Zheng Y. Zhang B. Wang R. Chen L. Wang Y. (2024). Untargeted serum and gastric metabolomics and network pharmacology analysis reveal the superior efficacy of zingiberis rhizoma recens-/euodiae fructus-processed coptidis rhizoma on gastric ulcer rats. J. Ethnopharmacol. 332, 118376. 10.1016/j.jep.2024.118376 38782310

