# Supplementary figures and images for: Standardized aqueous extract of Abutilon theophrasti Medic. ameliorates oxidative stress and inflammatory responses against hydrochloric acid/ethanol-induced gastric ulcer in rats
Source: Front Pharmacol. 2025 Jun 18;16:1599810. doi: 10.3389/fphar.2025.1599810 (PMC12213860; doi:10.3389/fphar.2025.1599810)

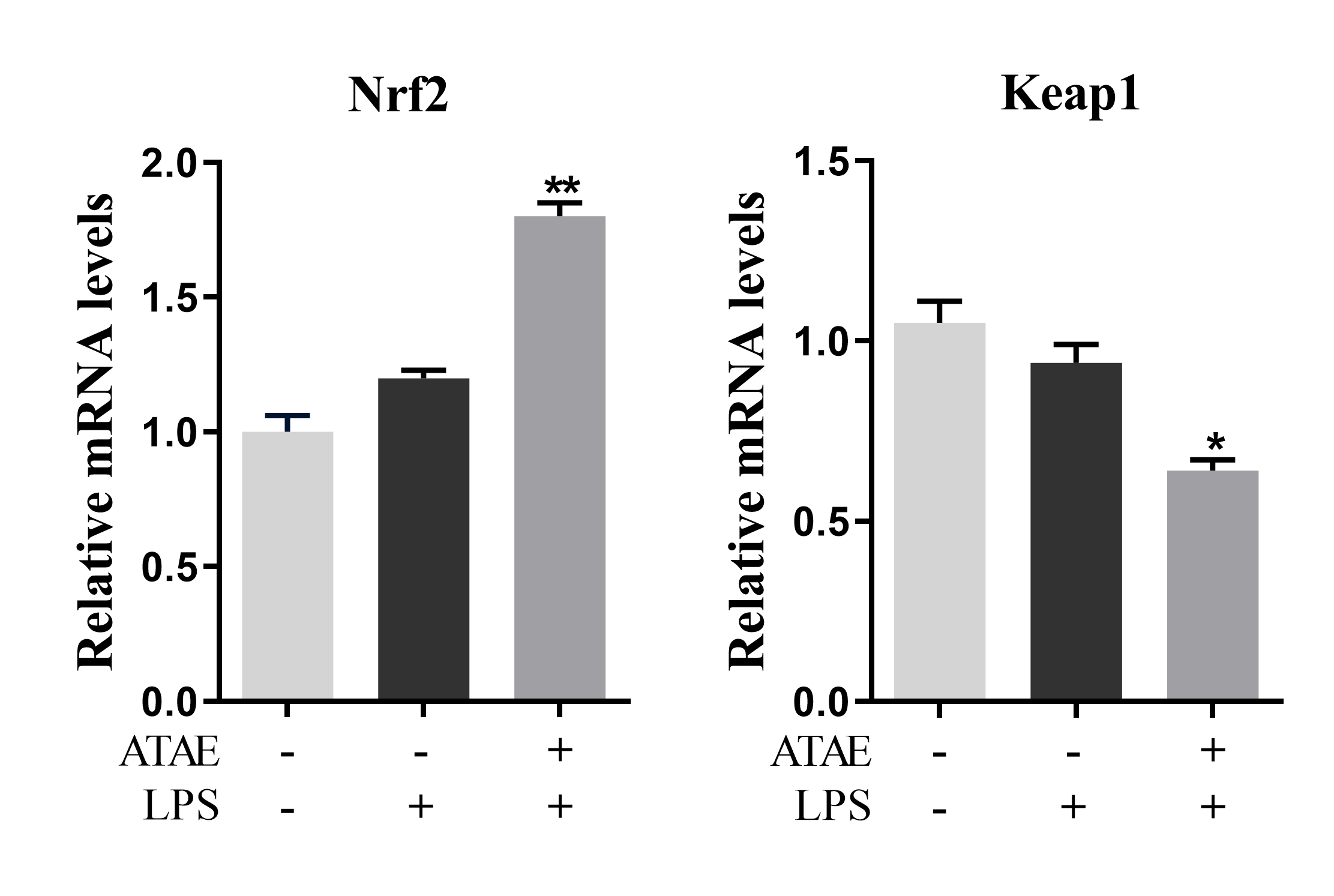

Supplement: Supplementary file 1 [file Image2.tif]

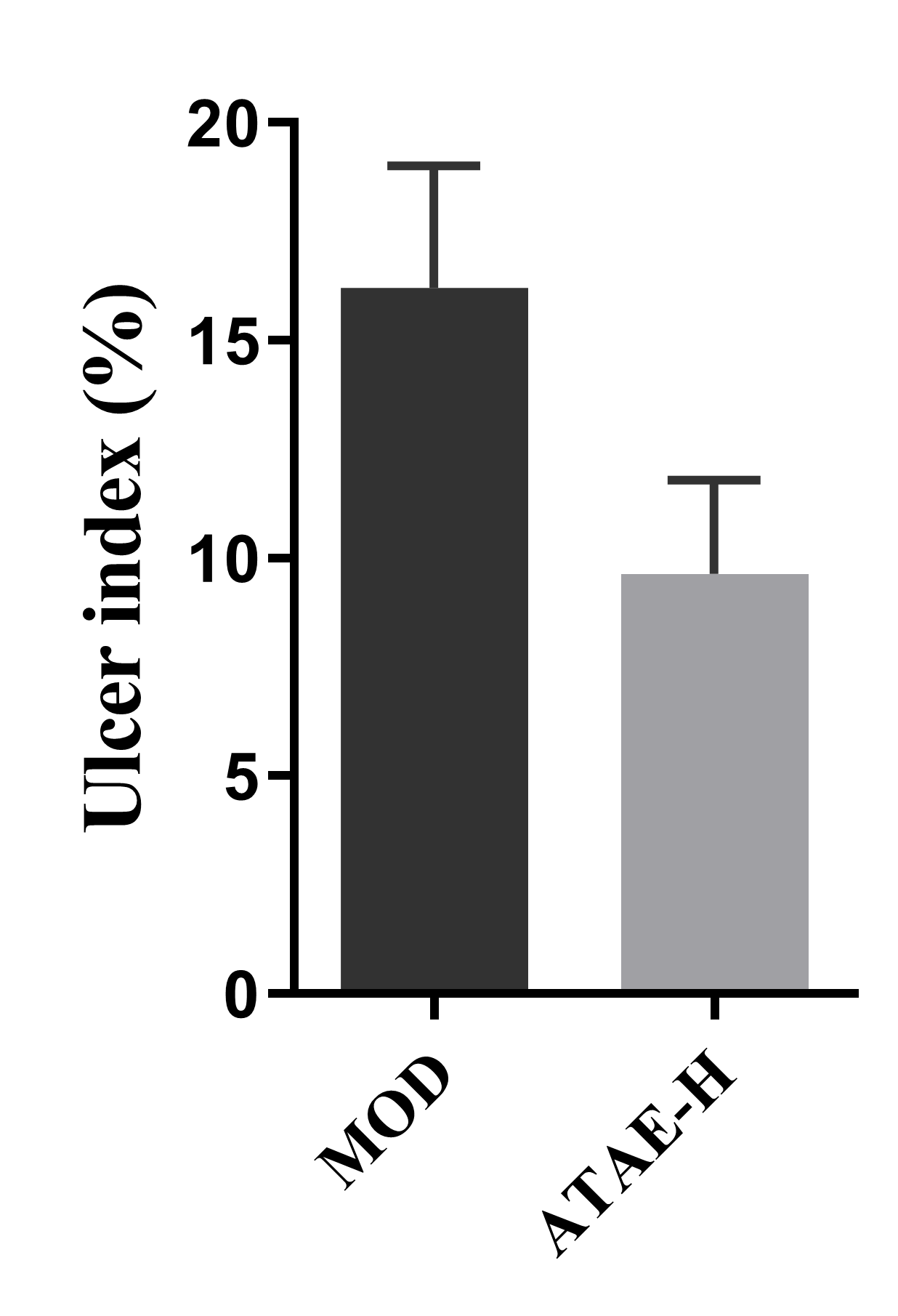

Supplement: Supplementary file 2 [file Image1.tif]
